# Gender equity and sustainable development through the lens of fertility intentions among highly educated women: a systematic review and meta-analysis

**DOI:** 10.1186/s12889-025-25842-y

**Published:** 2025-12-02

**Authors:** Ruobing Mei, Minghui Tan, Mengyun Liu, Leesa Lin

**Affiliations:** 1 Department of Infectious Disease Epidemiology, London School of Hygiene & Tropical Medicine, London, UK; 2 Laboratory of Data Discovery for Health Limited (D24H), Hong Kong, China; 3 Moonrise Initiative Limited, Hong Kong, China; 4 WHO Collaborating Centre for Infectious Disease Epidemiology and Control, School of Public Health, LKS Faculty of Medicine, The University of Hong Kong, Hong Kong, China; 5 The Institute of Social Medicine, School of Medicine, Zhejiang University, Hangzhou, China; 6 Population, Policy & Practice and Teaching Department, Institute of Child Health, University College London, London, UK

**Keywords:** Fertility intention, Childbearing intention, Fertility desire, Reproductive health, Female education, Meta-analysis

## Abstract

**Background:**

Declining fertility rates have raised concerns about potential population crises in many nations. While previous studies have established the link between female education and fertility intentions, no global meta-analysis has examined the fertility intentions of highly educated women.

**Methods:**

We searched Medline, Embase, Global Health, and APA PsycExtra for studies published between January 2000 and December 2022, without language restrictions. Eligible studies included cross-sectional, longitudinal, case-control, intervention, and qualitative studies that reported fertility intentions or influencing factors among females with at least a post-secondary education. We excluded abstracts, conference proceedings, letters, commentaries, editorials, reviews, and preprints. The pooled proportion of fertility intentions was estimated through a random-effects meta-analysis. We analyzed factors that influence fertility intentions using a data-based convergent synthesis design.

**Results:**

Out of the initial 4,804 studies identified, 35 studies were eligible for inclusion. The meta-analysis of fertility intentions included 17,292 highly educated women from 19 countries. The pooled proportion of highly educated women who did not expect children in the future was 12.2% (95% CI 8.4–16.7%), with substantial variation across regions, from 5.1% (2.6–8.3%) in the European Region to 22.9% (13.6–33.7%) in the Western Pacific Region. Factors associated with fertility intentions included not only the pursuit of a career and education (individual-level), the need for stable relationships and family support (family-level), but also (un)supportive environments (community-level), labor market and childcare services (institutional-level), and social norms and national policies (structural-level).

**Conclusion:**

Regional disparities in fertility intentions among highly educated women reflect underlying differences in social welfare systems, labor market structures, and gender norms. Policies aimed at supporting fertility in this group should move beyond a narrow focus on individual preferences and instead address the broader contextual factors that shape reproductive decision-making. Creating a supportive environment for parenting, through equitable leave entitlements, accessible childcare, and family-friendly employment policies, may contribute to fostering informed fertility choices among highly educated women.

**Trial registration:**

CRD42023404366.

**Supplementary Information:**

The online version contains supplementary material available at 10.1186/s12889-025-25842-y.

## Introduction

Gender equity and quality education are two core targets within the United Nations (UN) Sustainable Development Goals to be achieved by 2030 [1]. Over the past century, women’s educational attainment has increased substantially, playing a pivotal role in both social and human development [2, 3]. Investment in female human capital contributes significantly to economic growth [4], with evidence suggesting that increasing female labor force participation could boost gross domestic product by 5% in the United States of America and by 9% in Japan [5]. Studies also highlight the intergenerational transmission of advantages from increased female education to child health [6–8]. However, persistent gender inequity and systemic policy inadequacies, such as unpaid domestic responsibilities and limited parental leave, largely curtail female participation and impact across their lives [9, 10]. Addressing these challenges is crucial for unleashing women’s potential, catalyzing sustainable development, and positively influencing demographic trends, such as fertility rates.

Fertility rates have declined substantially worldwide over the past six decades, with the global average falling by 60% from 5.3 births per woman in 1963 to 2.3 in 2021 [11]. The population replacement rate, the total fertility rate (TFR) required to sustain a stable population, is estimated at 2.1 children per woman [12]. However, by 2017, the TFR had fallen below 2.0 in many areas of the world, including Europe, Australia, North America, East Asia, and Southeast Asia [13]. This decline in TFR has been particularly severe in countries such as the Republic of Korea, China, and Italy, where it has raised growing concerns about a national population crisis [14–16]. Projections suggest that by 2050, 151 of the 195 countries and territories studied may have TFRs below the replacement threshold [13], suggesting potential widespread population decline on a global scale.

Studies worldwide have shown a lower fertility rate in women with higher educational attainment [17, 18]. The reasons for this association are complicated and context-dependent [18], with several explanatory pathways proposed in the literature. First, a longer duration of education can lower the fertility rate by postponing marriage and childbirth [19, 20]. Second, more educated women may have better access to contraception and prenatal and neonatal care, which may contribute to both lower fertility and improved child survival [19–21]. Third, higher education is associated with an increased opportunity cost of childbearing in terms of lost income and career advancement [19, 21]. In addition, external factors such as social expectations of motherhood, policy support, and political stability may also influence the fertility intentions of highly educated women [22].

Given the critical role of female education in driving social and human development, understanding the fertility intentions of highly educated women is crucial for uncovering gaps and informing policies that eliminate obstacles towards the Sustainable Development Goals. While previous studies have attempted to summarize evidence on fertility intentions in this population [23–25], they largely focused on qualitative rationales and have not provided a comprehensive assessment of the magnitude of fertility intentions. There has been a lack of effort to quantitatively synthesize global evidence on fertility intentions among women with higher education. This systematic review and meta-analysis aims to quantify the magnitude of fertility intentions among women with higher education in low-fertility countries and explores the key concerns and barriers influencing their fertility decisions.

## Methods

### Study design

We conducted the study in adherence to the Preferred Reporting Items for Systematic Reviews and Meta-Analyses statement (PRISMA) [26]. The protocol of the systematic review was registered with PROSPERO (CRD42023404366).

### Search strategy

The literature search was performed on Medline, Embase, Global Health, and APA PsycExtra for studies published between January 2000 and December 2022, without language restrictions. Non-English studies were translated into English using Google Translate or by multilingual researchers. Relevant search terms were used to identify investigations of women’s fertility attitudes, behaviors, or awareness in countries with a below-replacement fertility rate of 2.1 births per woman [27]. The full search strategy is detailed in Table S1.

### Eligibility criteria and selection process

The inclusion and exclusion criteria used at all stages of the study can be found in Table S2. Cross-sectional, longitudinal, case-control, intervention, and qualitative studies were included, whereas abstracts, conference proceedings, letters, commentaries, editorials, reviews, and preprints were excluded. Studies were included if the study population met all the following inclusion criteria: [1] women who were nulliparous; [2] women who obtained at least post-secondary education (above 12 years of schooling), including universities, (community) colleges, vocational schools, and technical training institutes; [3] women who resided in countries with a below-replacement fertility rate of 2.1 births per woman; [4] women of reproductive age between 15 and 49 years. Due to the lack of standardized scales for assessing fertility intentions, we focused on the fertility intentions of highly educated women as reported at the time of data collection in each included study. We measured their future fertility intentions at that time and did not set restrictions on first or subsequent childbearing. Two reviewers (RM and MT) independently screened the titles and abstracts of the retrieved records, followed by full-text review. All disagreements were first resolved through mutual discussion, and further disputes were addressed by a third reviewer (ML). We quantified the inter-coder reliability for the selection process using Cohen’s kappa coefficient.

### Data extraction and quality assessment

For eligible studies, we extracted data on the year of publication, country, study design, study setting, study period, data source, sample size, missingness, participant characteristics (including age, ethnicity, education, and marital status), fertility intentions, and reasons or conditions related to fertility intentions. For studies with longitudinal data, we extracted the baseline estimate of fertility intentions. We developed a risk of bias assessment tool based on existing tools, including the Newcastle-Ottawa Scale [28] and the Strengthening the Reporting of Observational Studies in Epidemiology (STROBE) statement [29]. We comprehensively assessed the risk of bias in the studies across four domains: representativeness, sample size, non-respondents, and data collection tool. Each component was assigned a rating of low risk, high risk, or unclear. Data extraction and risk of bias assessment were conducted by one reviewer (RM or MT) and were subsequently cross-checked by another reviewer. Any discrepancy was resolved through discussion to reach a consensus.

### Data synthesis and analysis

The main outcome measure was the proportion of highly educated women who did not intend to have children. For included studies evaluating the importance of specific conditions for women’s decision to start motherhood, the proportions rated for each condition were examined. Statistical heterogeneity of the pooled estimates was assessed via the $\:{I}^{2}$ statistic. We synthesized the proportions of the outcomes using the DerSimonian-Laird random-effects meta-analyses, with a Freeman-Tukey double arcsine transformation due to a high level of heterogeneity [30, 31]. The confidence interval around the pooled effect was calculated using Newcombe’s asymptotic method with continuity correction. A sensitivity analysis was performed to assess the robustness of the meta-analysis results using the leave-one-out analysis.

We performed subgroup analyses by study location [classified by the World Health Organization (WHO) region], income classification of the country [high-income country or lower-/upper-middle-income country based on the World Bank classification], study period [before 2005, 2005–2014, 2015–2018 or 2019 and after], mean age of participants [$\:<$ 25 years or $\:\ge\:$ 25 years], recruitment setting [school, household, or multi-center], and sample size [below 200, 200–500, or above 500]. In addition, to analyze the effect of leave policies on the intentions not to have children among highly educated women, we categorized the studies based on the presence of national paid maternity, paternity, and/or parental leave policies in effect at the time each study was conducted. We explored sources of heterogeneity through meta-regressions using the metafor package. The covariates in the meta-regressions included study location, country income classification, study period, age group, recruitment setting, study type, participant sample size, and leave policy. Publication bias was assessed through the funnel plot and Egger’s regression test. A p-value of less than 0.05 was considered statistically significant. All analyses and visualizations were done using R (version 4.3.1).

The factors related to fertility intentions were analyzed using a data-based convergent synthesis design [32]. This design involves converting quantitative data into qualitative formats and analyzing them alongside qualitative and mixed-methods data. Two reviewers (MT and RM) independently extracted messages and information from the included studies and repeated the process for all studies to generate a list of themes and codes, based on the socio-ecological model – a commonly used and effective approach to describing the complex process of fertility intentions, which involves five analytical themes: [1] individual level, [2] family level, [3] community level, [4] institutional level, and [5] structural level [33, 34]. Differences were resolved through discussion and, if required, a third reviewer (ML) was consulted to achieve consensus. These codes were subsequently refined through comparison with other studies and team discussion, leading to adjustments and reclassification into different categories.

## Results

### Study search and characteristics

**Fig. 1:**
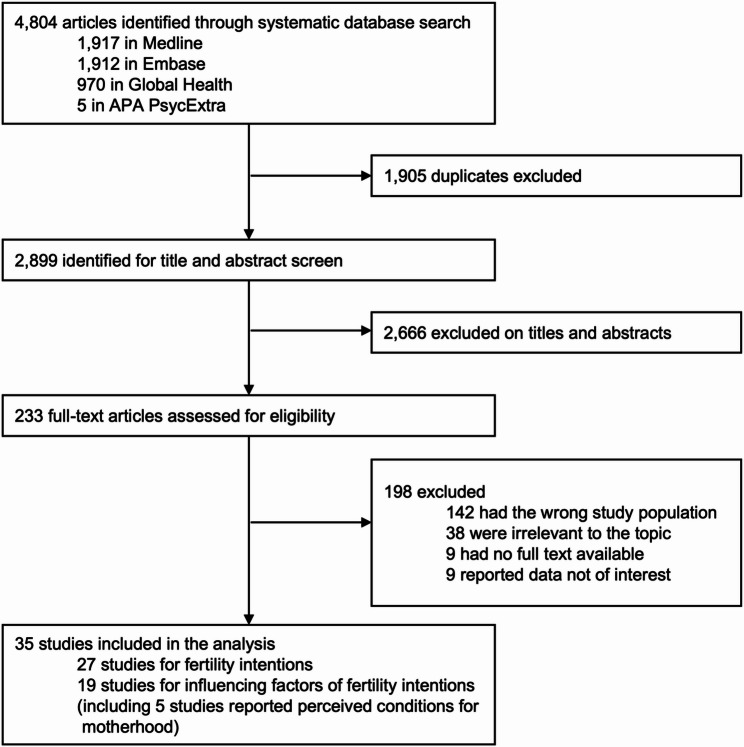
Flow diagram of study selection

The initial search identified 4,804 studies from four databases, and 2,899 remained after de-duplication for title and abstract screening, of which 233 were assessed for full-text review. We retained 35 studies for final analysis, of which 27 reported data on fertility intentions, and 19 reported influencing factors related to fertility intentions (Fig. 1; Table 1). The inter-coder reliability revealed considerable agreement between the reviewers, with Cohen’s kappa statistics above 0.9 at all stages. All included studies except two (one longitudinal and one case-control study) used a cross-sectional design. Quality assessment is presented in Table S3.

**Table 1:** Characteristics of included studies

Study	Study period	Country	Income classification	Study design	Recruitment setting	Sample size	Degree	Inclusion in research questions		Proportion of intentions not to have children
								Fertility intentions	Influencing factors of fertility intentions	
Benzies (2006)	NA	Canada	HIC	Qualitative study	Multiple settings	11	Not specified	No	Yes	NA
Berrington (2014)	1981	United Kingdom	HIC	Quantitative study (longitudinal)	Multiple settings	571	Bachelor	Yes	No	8.4%
Birch Petersen (2016)	2014	Denmark	HIC	Qualitative study	Health center	22	Master	No	Yes	NA
Chan (2015)	2013	China	LMIC	Quantitative study (cross-sectional)	School	275	Bachelor	Yes	Yes	20.0%
Delbaere (2021)	2017–2018	Sweden, Belgium, Greece	HIC	Quantitative study (cross-sectional)	School	406	Bachelor	Yes	No	4.3%
Grace (2022)	2017	United Kingdom	HIC	Mixed-methods study	Communication channel	20	Bachelor	No	Yes	NA
Hickman (2018)	NA	United States of America	HIC	Quantitative study (cross-sectional)	School	512	Master	Yes	Yes	23.4%
Lampic (2006)	2004	Sweden	HIC	Quantitative study (cross-sectional)	School	222	Bachelor	Yes	Yes	4.0%
Lee (2021)	2018	Republic of Korea	HIC	Quantitative study (cross-sectional)	School	563	Bachelor	Yes	No	50.4%
Machado (2014)	2012	Portugal	HIC	Quantitative study (cross-sectional)	School	3585	Bachelor	No	Yes	NA
Nouri (2014)	2012	Austria	HIC	Quantitative study (case-control)	School	170	Bachelor	Yes	No	5.9%
Ozerdogan (2018)	2015	Turkey	LMIC	Quantitative study (cross-sectional)	School	262	Bachelor	Yes	No	0.4%
Peterson (2012)	NA	United States of America	HIC	Quantitative study (cross-sectional)	School	138	Bachelor	Yes	No	15.9%
Place (2020)	2017–2018	Mexico	LMIC	Quantitative study (cross-sectional)	School	228	Not specified	Yes	No	52.2%
Prior (2019)	2017	Australia	HIC	Quantitative study (cross-sectional)	School	930	Bachelor, Master, PhD	Yes	No	7.6%
Safari-Faramani (2018)	2015–2016	Iran	LMIC	Qualitative study	School	11	PhD	No	Yes	NA
Shin (2020)	2019	Republic of Korea	HIC	Quantitative study (cross-sectional)	School	85	Bachelor	Yes	Yes	30.6%
Skoog Svanberg (2006)	2004	Sweden	HIC	Quantitative study (cross-sectional)	School	200	Master	Yes	Yes	6.0%
Sørensen (2016)	2016	Denmark	HIC	Quantitative study (cross-sectional)	School	381	Bachelor	Yes	No	3.3%
Stack (2020)	2017	United States of America	HIC	Quantitative study (cross-sectional)	School	1537	Master, PhD	No	Yes	NA
Tan (2011)	NA	Singapore	HIC	Quantitative study (cross-sectional)	Community	127	Bachelor, Master	Yes	Yes	29.5%
Tydén (2006)	2004	Sweden	HIC	Quantitative study (cross-sectional)	School	324	Bachelor, Master, PhD	Yes	Yes	2.7%
Virtala (2011)	2008	Finland	HIC	Quantitative study (cross-sectional)	School	2980	Not specified	Yes	No	5.9%
Vujčić (2017)	2016	Republic of Serbia	LMIC	Quantitative study (cross-sectional)	School	271	Not specified	Yes	Yes	4.8%
Xu (2022)	2020	China	LMIC	Quantitative study (cross-sectional)	School	379	Not specified	Yes	Yes	33.8%
Zhang (2022)	2021	China	LMIC	Quantitative study (cross-sectional)	School	4025	Bachelor, Master, PhD	Yes	Yes	18.6%
Rosina (2009)	2003	Italy	HIC	Quantitative study (cross-sectional)	Community	214	Bachelor	Yes	No	20.2%
Araujo (2020)	2019	Brazil	LMIC	Quantitative study (cross-sectional)	School	155	Bachelor	Yes	Yes	13.1%
Barron (2022)	NA	United States of America	HIC	Quantitative study (cross-sectional)	School	2146	Not specified	Yes	No	7.6%
Bretherick (2010)	2006	Canada	HIC	Quantitative study (cross-sectional)	School	360	Bachelor	Yes	No	11.1%
Brinton (2018)	2012	Japan, Spain, Sweden, United States of America	HIC	Qualitative study	Multiple settings	207	Bachelor	No	Yes	NA
Cheung (2019)	2016	Australia	HIC	Quantitative study (cross-sectional)	Multiple settings	120	Not specified	Yes	No	5.0%
Mogilevkina (2016)	2012	Ukraine	LMIC	Quantitative study (cross-sectional)	School	858	Not specified	Yes	No	16.1%
Rovei (2010)	2006–2007	Italy	HIC	Quantitative study (cross-sectional)	School	607	Not specified	Yes	No	0.0%
Spence (2016)	2012	United States of America	HIC	Qualitative study	School	11	Not specified	No	Yes	NA

*HIC* High-income country, *LMIC* Low-and-middle-income country, *NA* Not available

### Fertility intentions of highly educated women

The 27 studies included a total of 17,292 highly educated women. Studies were from 19 countries in the three WHO regions: eleven in the European Region (EUR), four in the Region of the Americas (AMR), and four in the Western Pacific Region (WPR). No studies were conducted in the Eastern Mediterranean Region (EMR), African Region (AFR), or South-East Asia Region (SEAR). Of these, 20 studies (74.1%) were from high-income countries, and 7 studies (25.9%) were from lower- or upper-middle-income countries. Most countries contributed only one or two studies, while China and the United States of America each had three studies, and Sweden had four studies. Four studies (14.8%) were from countries without a national leave scheme during the study period.

The included studies were published between 2006 and 2022, with study periods spanning from 1981 to 2021. The mean age of participants was 23.0 years, ranging from 20.2 to 30.7 years across studies. Sample sizes varied from 85 to 4,025 women. Two studies were community-based, two studies recruited participants from different types of settings, and the others were school-based (*n* = 23, 85.2%). According to self-reported data, 43.8% of participants held a bachelor’s degree (*N* = 7,576), 12.0% had a graduate degree (*N* = 2,075), while the remaining (*N* = 7,641, 44.2%) had received higher education but no information on the specific level was reported.

**Fig. 2:**
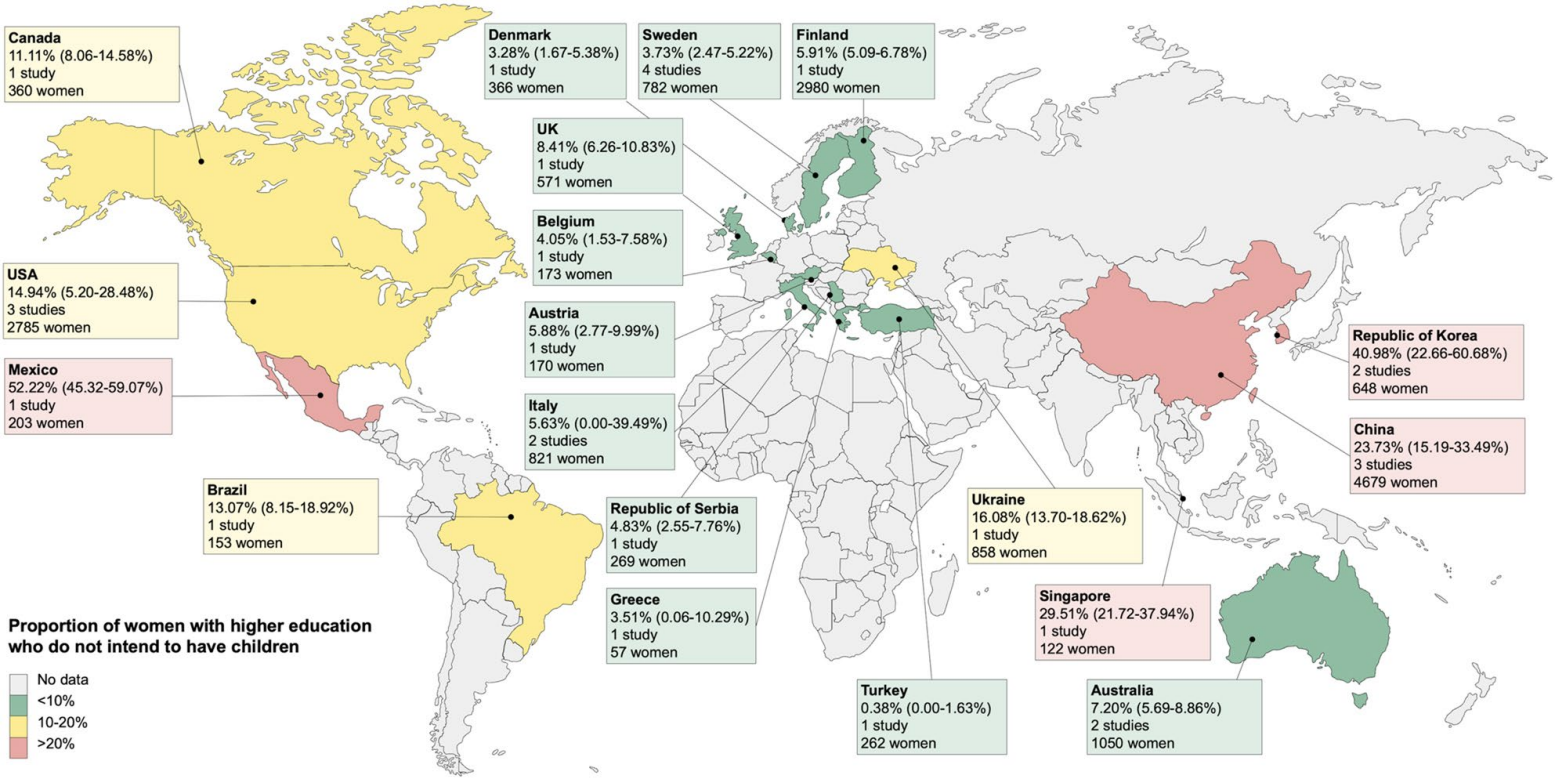
Country-specific proportions of highly educated women who intend not to have children. Fertility intentions were reported in studies conducted in each country. Pooled proportions were calculated if the country had more than one study. Map lines delineate study areas and do not necessarily depict accepted national boundaries

The overall proportion of highly educated women who did not intend to have children was 12.2% (95% CI 8.4–16.7%), with substantial between-study heterogeneity ($\:{I}^{2}$ ranged from 98.2 to 98.7%, *p* < 0.0001). The proportion varied substantially across WHO regions (Table 2): women in the WPR reported the highest level of intentions not to have children (22.9%, 13.6–33.7%, *n* = 8), followed by the AMR (19.0%, 9.1–31.5%, *n* = 6). The lowest proportion of women lacking the desire for children was found in the EUR (5.1%, 2.6–8.3%, *n* = 13). Considerable within-region variations were observed in all three regions (Figs. 2 and S1). Country-specific estimates were mostly based on one or two studies. In the AMR and WPR, the intentions not to have children were reported as highest in Mexico (52.2%, 45.3–59.1%, *n* = 1) and the Republic of Korea (41.0%, 22.7–60.7%, *n* = 2) respectively, almost five times higher than their lowest counterparts such as Canada (11.1%, 8.1–14.6%, *n* = 1) and Australia (7.2%, 5.7–8.9%, *n* = 2).

**Table 2:** Subgroup analyses of highly educated women who intend not to have children

Subgroup	No. of studies	Proportion (%)	95% CI (%)	Cochran’s Q	(%)	Subgroup differences (*p*-value)
Overall	27	12.24	8.36–16.73	1703.63	98.5	
**Study location**						**< 0.0001**
Region of the Americas	6	19.01	9.07–31.49	262.85	98.1	
European Region	13	5.09	2.60–8.32	313.84	96.2	
Western Pacific Region	8	22.87	13.61–33.69	443.59	98.4	
**Income classification**						0.24
High income countries	20	10.79	6.65–15.76	1049.72	98.2	
Low-and-middle-income countries	7	16.74	8.68–26.75	354.85	98.3	
**Study period** ^*^						**0.016**
Before 2005	5	7.30	2.98–13.25	49.94	92.0	
2005–2014	6	8.01	2.82–15.48	278.01	98.2	
2015–2018	8	11.67	2.29–26.69	796.42	99.1	
2019 and after	4	23.31	14.75–33.13	52.49	94.3	
NA	4	18.10	7.94–31.20	116.21	97.4	
**Age of participants**						0.11
< 25 years	22	11.17	7.06–16.06	1608.61	98.7	
≥ 25 years	5	17.72	11.16–25.38	34.27	88.3	
**Recruitment setting**						**< 0.0001**
School	23	11.89	7.69–16.84	1653.49	98.7	
Community	2	24.29	15.69–34.07	3.74	73.3	
Multiple settings	2	7.31	4.68–10.46	1.52	34.1	
**Sample size**						0.89
Below 200	7	13.61	7.05–21.83	63.18	90.5	
200–500	11	11.13	4.46–20.26	527.70	98.1	
Above 500	9	12.61	6.48–20.39	1107.51	99.3	
**Leave policy** ^†^						**0.0002**
No leave scheme	4	13.09	6.58–21.37	88.92	96.6	
Maternity leave only	4	13.64	0.71–38.07	271.38	98.9	
Maternity and paternity leave, without parental leave	7	25.33	12.46–40.87	534.38	98.9	
Maternity and parental leave, without paternity leave	3	11.02	6.15–17.06	17.22	88.4	
Paternity and parental leave, without maternity leave	5	4.92	2.91–7.40	12.97	69.2	
All leave schemes (maternity, paternity, and parental)	4	4.74	3.53–6.12	6.28	52.2	

Maternity leave refers to job-protected paid leave for employed women before and after childbirth. Paternity leave refers to job-protected paid leave for employed men following childbirth. Parental leave refers to job-protected leave available to employed parents, typically taken after maternity or paternity leave, and may be used by both parents either consecutively or simultaneously. NA = not available

^*^For studies covering multiple years, the earliest year of data collection was used as the reference point

^†^Leave policies refer to national paid leave policies sourced from the World Bank [35]

Table 2 presents the results of the subgroup analysis. When stratified by study period, the pooled proportion of women who did not intend to have children increased over time, from 7.3% (95% CI 3.0–13.3.0.3%, *n* = 5) before 2005 to 11.7% (2.3–26.7%, *n* = 8) between 2015 and 2018, and reached 23.3% (14.8–33.1%, *n* = 4) after 2018. The proportion was higher in community-based studies (24.3%, 15.7–34.1%, *n* = 2) than in those conducted in schools (11.9%, 7.7–16.8%, *n* = 23) and in multiple types of settings (7.3%, 4.7–10.5%, *n* = 2). No significant differences in fertility intentions among highly educated women were found based on sample size and country income classification.

A significant difference in fertility intentions was observed across studies with different types of national paid leave schemes (*p* = 0.0002). Studies from countries with national paid parental leave reported lower proportions of highly educated women intending not to have children: 4.7% (95% CI 3.5–6.1%, *n* = 4) in countries offering all three types of leave, and 4.9% (2.9–7.4%, *n* = 5) in countries with paternity and parental leave but no maternity leave. In contrast, the highest proportion was found in studies from countries without paid parental leave, even when paid maternity and paternity leave were available (25.3%, 12.5–40.9%, *n* = 7).

Residual heterogeneity remained high ($\:{I}^{2}$=97.1%, *p* < 0.0001) after including all covariates in the multivariable meta-regression model, indicating substantial heterogeneity between populations, most of which could not be accounted for by the covariates (Table S4). The sensitivity analysis (Figure S2) showed minor changes when excluding a single study at a time, with the pooled proportion ranging from 11.1% (95% CI 7.8–15.0%) to 13.1% (9.3–17.5%). The funnel plot (Figure S3) and Egger’s regression test (*p* = 0.86, Table S5) showed no evidence of publication bias.

### Factors influencing fertility intentions of highly educated women

#### Quantitative synthesis

**Fig. 3:**
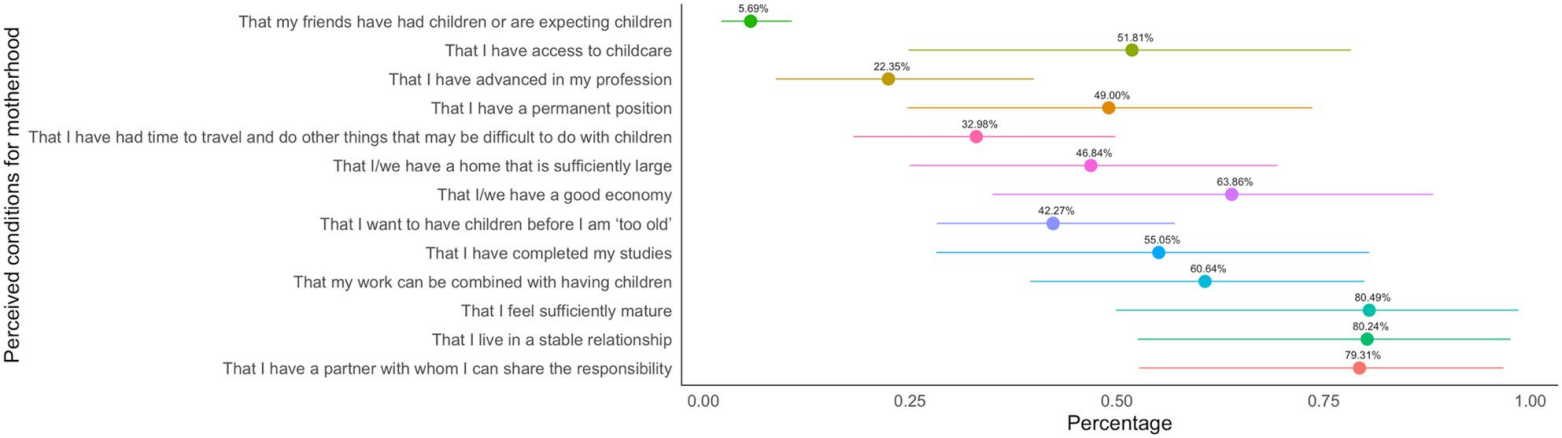
Pooled proportions of perceived conditions by highly educated women for their decisions to become motherhood. The pooled proportions indicate the percentage of participants who rated each condition as either “very important” or “important”. The points refer to the pooled proportions from five studies, and the lines centered on the points represent the 95% CIs

Among the 19 studies examining factors influencing fertility intentions of highly educated women, 10 studies [36–45] reported results on perceived conditions for motherhood using part of the Swedish Fertility Awareness Questionnaire (13 items) [37], but only five from Sweden, the Republic of Korea, and China that reported numerical data [36–39, 41] were included in the meta-analysis. Participants were asked to rate each item, from “not important at all” to “very important” (five choices), on each item in the questionnaire. Figure 3 summarizes the proportions of participants who rated either “very important” or “important” for each condition. The conditions women considered the most important for motherhood were “I feel sufficiently mature” (80.5%, 95% CI 49.8–98.5%), “I live in a stable relationship” (80.2%, 52.5–97.6%), “I have a partner with whom I can share the responsibility” (79.3%, 52.7–96.8%), “I/we have a good economy” (63.9%, 34.9–88.2%), and “My work can be combined with having children” (60.6%, 39.5–79.9%).

#### Qualitative synthesis

In addition to the perceived conditions for motherhood, fifteen studies (nine quantitative, five qualitative, and one mixed-methods) from twelve countries provided data on factors influencing fertility intentions among highly educated women, two-thirds (*n* = 10) of which were from high-income countries. Nearly one-third of the studies were conducted in each of the three WHO regions: AMR (*n* = 6), EUR (*n* = 5), and WPR (*n* = 5), while only one study took place in the EMR. All included studies were identified, analyzed, and synthesized qualitatively to examine factors associated with fertility intentions based on the socio-ecological model. The qualitative synthesis is detailed in the appendix (Figure S4, Tables S6-7).

##### Individual level

Individual factors associated with fertility intentions identified by both qualitative and quantitative studies included socio-demographic characteristics (including age and education), career pursuits, financial and lifestyle considerations, emotional and psychological readiness, personal preferences and attitudes, and knowledge about reproductive health services. Additionally, qualitative research highlighted the role of religious factors in shaping these intentions.

##### Family level

Both quantitative and qualitative studies mentioned that relationship stability and partners’ fertility intentions were related to women’s fertility intentions. Quantitative studies focused on a lack of family support as an objective factor. However, qualitative research offered additional insights into highly gender-unequal household division of labor.

##### Community level

Both quantitative and qualitative studies identified high workload as a significant barrier to fertility intentions. Two qualitative studies indicated that highly educated women found it challenging to balance work and family [46, 47]. Parental leave burden on colleagues, career threat, and lack of childbearing-supportive programs were mentioned as reasons for delaying childbearing among female residents in a quantitative study [48]. Qualitative research added that work competition, employer prejudice toward women with caregiving responsibilities, and the risk of incurring a motherhood wage penalty might discourage fertility intentions.

##### Institutional level

Both quantitative and qualitative studies emphasized the importance of labor market stability and childcare services. A supportive labor market with reduced working hours and an enhanced welfare system offering proper leave policies and wage replacement benefits might be strong incentives to support fertility decisions. However, concerns about housing and childrearing responsibilities, particularly the accessibility, affordability, and quality of education and childcare services, were prevalent in countries that lacked government-subsidized services.

##### Structural level

All factors at the structural level came from qualitative research. In addition to macro-level stressors, including economy, environment, and migration [47, 49, 50], social norms centered on gender ideology intensified society-induced expectations for women’s roles [46, 47, 50, 51]. The absence of supportive social structures and policy support exacerbated challenges in maternal training and failed to effectively address work-family conflicts.

## Discussion

This systematic review synthesized available data on the levels of and reasons for fertility intentions among highly educated women in low-fertility countries. On average, one in eight women in this population reported no intention to have children. Fertility intentions varied across regions, with the highest proportions of highly educated women intending not to have children reported in the Western Pacific and the Americas. Women residing in countries with comprehensive national paid parental leave schemes were less likely to express intentions to remain childless. The qualitative analysis suggests that factors across multiple levels, from individual readiness and family support to the working environment and broader social welfare systems, affect the fertility intentions of highly educated women. The findings from the qualitative analysis also highlight the important role of gender norms and ideologies in shaping women’s reproductive planning, including their partners’ fertility intentions and contributions to domestic work, and prevailing gender-inequitable social norms in the workplace and society at large [47, 50, 51].

The proportion of highly educated women intending not to have children varied from 5.1% (95% CI, 2.6–8.3%) in the EUR to 22.9% (13.6–33.7%) in the WPR. Though these estimates were based on studies from a limited number of countries in each region (four countries in WPR, four in AMR, and 11 in EUR), they were robust in the leave-one-out sensitivity analysis and mirrored regional trends in actual fertility rates [27]. The large regional variations underscore the role of sociocultural, economic, and policy environments in shaping reproductive preferences. In Europe, comprehensive welfare systems offering gender-equitable leave, subsidized childcare, and flexible workplace arrangements likely support childbearing among highly educated women [52]. In contrast, countries in the WPR – particularly the Republic of Korea, Singapore, and China – often face structural barriers, including long working hours, maternity wage penalties, inadequate paid leave, limited childcare support, and traditional gender norms that place disproportionate childcare and household responsibilities on women, all of which may discourage fertility among women with high educational and career aspirations [52–55]. No studies from the AFR and SEAR regions were included, likely reflecting the higher fertility rates and smaller populations of highly educated women in these regions.

In addition to the factors examined in the meta-regressions, the observed between- and within-country variations in fertility intentions may also be attributed to differences in other population characteristics and survey designs. For example, in Italy, a national household survey estimated that 20.2% of married women with higher education, primarily aged above 30, reported no intention to have children [56], while nearly all female students in a university-based survey, with a mean age of 21.9 years, expressed the intention to do so [44]. The discrepancy in fertility intentions between the two studies is probably due to the selected population that childless, married women are more likely to remain low fertility intentions and may be influenced by their partners’ preferences in family decisions compared with their younger peers [57]. The discrepancy highlights the importance of taking into account population characteristics and survey designs when interpreting fertility intentions. Furthermore, we observed substantial heterogeneity in the meta-analysis estimates, which was not fully explained by the covariates included in the meta-regression models. We speculate that systemic factors, such as institutional and sociocultural influences described in our qualitative results, may also contribute to the observed heterogeneity.

Our analysis revealed a significant difference in fertility intentions based on the availability of national paid leave policies: 13.1% of highly educated women who lived in countries without any leave schemes reported intentions not to have children, compared with 4.7% in those with all three types of leave policies. The findings align with previous evidence on the positive impact of paid leave policies on fertility in low-fertility settings [58]. Comprehensive leave schemes reduce the career and financial trade-offs associated with childbearing while promoting gender equity by enabling greater paternal engagement [59–61]. In countries offering maternity and paternity leave, the share of women intending not to have children was significantly higher (25.3%) than in those also offering parental leave (4.7%), highlighting the essential role of gender-neutral policies that support shared caregiving responsibilities. Parental leave has been shown to facilitate sustained paternal involvement beyond the early postpartum period, which helps mitigate the disproportionate caregiving burden on mothers [59, 60]. Empirical evidence from Europe indicates that fathers’ uptake of paid leave increases their involvement in childcare, including feeding, bathing, and bedtime routines, contributing tangibly to daily caregiving [61]. These contributions extend beyond childcare to include shared responsibility for household labor, physical and mental support during pregnancy and the postpartum period, and active participation in antenatal and postnatal care [52, 62]. Evidence from the Generations and Gender Survey shows that men’s engagement in housework has a stronger positive effect on women’s fertility intentions than childcare involvement alone [52, 63, 64]. The findings underscore the importance of policies that promote a more gender-balanced division of family work. By enabling and normalizing men’s caregiving contributions, these policies can foster a gender-egalitarian equilibrium that supports fertility realization, particularly among highly educated women navigating competing professional and family aspirations.

Our qualitative synthesis, structured around the socio-ecological model, revealed that the multilevel barriers to fertility essentially reflect deep-seated structural contradictions encountered by highly educated women in integrating reproductive autonomy with social roles. Drawing on Sen’s capability approach [65, 66], the current social environment systematically restricts women’s substantive freedom to transform personal resources into fulfilling their reproductive wishes. The occupational penalties, unequal distribution of family responsibilities, and limited availability of childcare resources constitute a deprivation of reproductive capability. Fraser’s redistribution-recognition paradigm further provides a dual lens for analyzing gender-based barriers [67]. First, workplace penalties and economic pressures reflect the devaluation of reproductive labor in the market economy. However, existing policies fail to recognize and compensate for childbearing and childcare as socially necessary reproductive labor, resulting in the privatization of childcare costs. Second, the persistence of gendered roles within households and workplace cultural biases constitutes institutional misrecognition of women’s agency. Promoting reproductive autonomy among highly educated women requires reframing childbirth from “individual risk” to “shared social responsibility”.

Addressing the multifaceted barriers to childbearing requires coordinated, multisectoral policy interventions and collaboration between the public sector and industry stakeholders. First, from the perspective of individual needs, women’s decisions to delay or refuse fertility are mainly associated with educational, career, and financial factors. Since unmatched life planning between ages and personal pursuits can increase the risk of age-related infertility, it is crucial to monitor and sustain fertility health through preventive care, including public education programs to improve reproductive awareness, encourage proactive fertility health screening, and provide family planning counseling [68, 69]. Second, in terms of the family unit, attitudinal alignment between women and their partners is an important determinant of fertility decisions. Since childbearing and childrearing are shared endeavors between men and women, men should provide essential support and share family responsibilities, mitigating gender disparities in the division of household labor and easing the domestic care burden on women [63]. Furthermore, career penalties in the workplace and unsupportive colleagues were commonly cited as factors at the community level. Equitable and supportive measures, including mental health support workshops and flexible maternity care arrangements, are encouraged to uphold women’s rights in the workplace [70]. Implementing flexible working arrangements, such as remote or hybrid options, part-time schedules, and flexible working hours, can enable highly educated women to balance professional and household responsibilities more effectively [71–73]. Considering macro and systemic factors, the labor market and childcare services can also affect the fertility intentions of highly educated women. Expanding access to affordable, equitable, and quality childcare services is crucial to reducing the childcare burden on families and supporting childrearing [12]. Lastly, governments should formulate robust and effective national policies that address the contextual factors contributing to fertility decline, such as national paid leave entitlements, to ease financial stress and encourage shared caregiving responsibilities in dual-earner households [74].

However, we must emphasize that proposed policy interventions should operate at the intersection of reproductive autonomy and societal support. While expanding fertility support systems, policies should rigorously avoid prescriptive pronatalism that constrains women’s self-determination. True autonomy necessitates eliminating structural barriers to enable informed choices – not steering decisions. Simultaneously, policies must redistribute care labor costs and deconstruct gendered norms equating women’s worth with motherhood. Crucially, interventions must distinguish between enabling conditions (supporting desired fertility) and coercive incentives (imposing fertility expectations). Maintaining this equilibrium is essential to upholding the principles of reproductive justice – that is, empowering individual choice while collectively mitigating structural constraints.

This study focused on the fertility intentions of highly educated women in low-fertility countries; therefore, the results should not be extrapolated to other populations or settings. Since most of the included studies were school-based (*n* = 23, 85.2%), the pooled estimates mainly reflect the intentions of young women. There are a few limitations that warrant consideration. First, while the analyses were based on data from 19 countries with diverse sociocultural norms and policy environments, the limited geographic scope may constrain the generalizability of the findings. Second, most countries contributed only one or two studies, and variation existed across studies in terms of study period, population characteristics, and survey design, which may limit the validity of direct cross-country comparisons. However, the leave-one-out analysis indicated that the overall results were robust and not disproportionately influenced by any single study. Third, we acknowledge that the transformation of quantitative data into qualitative themes may entail a loss of statistical precision, which is an inherent limitation of the data-based convergent synthesis design; however, for this exploratory research, the benefits of achieving an integrated conceptual understanding outweigh this limitation. Fourth, fertility intentions have temporal dynamics and may not align with eventual fertility behaviors. Longitudinal or register-based studies that track individual-level fertility trajectories over time are needed to better understand the intention-behavior gap in low-fertility contexts. In addition, while studies included in the review did not have a standardized instrument for assessing fertility intentions, evaluating the share of highly educated women who did not intend to have children provided valuable insights despite slight data variability. Future studies that establish a standardized framework for analyzing fertility intentions are needed to guide fertility health research. Finally, the study examined the effects of national leave policies on fertility intentions among highly educated women as a contextual factor. Future investigations could benefit from a more comprehensive analysis of policy design, including the duration, eligibility, generosity, and inclusiveness, to further illuminate their effects on women’s fertility planning.

## Conclusion

In conclusion, we have reviewed the evidence to highlight that fostering fertility support for highly educated women may contribute to advancing gender equity and promoting sustainable development. While most highly educated women did not intend to be childless, timely policy interventions, such as paid leave entitlements and accessible, affordable childcare services, are warranted to enable informed fertility decisions while upholding reproductive autonomy. With evolving gender norms and significant transformations in women’s roles, paralleled by shifts in work patterns, the contribution of women to society, the economy, and global progress has never been more critical. To harness this skilled talent pool effectively, it is essential to reassess and modernize family policies to align with these significant societal changes. As an integral issue of gender equity during the progress towards sustainable human development, the global challenge of fertility calls for collective efforts from inclusive communities, gender-egalitarian structures, and supportive systems, including flexible work arrangements, equitable employment practices, and family-oriented social welfare schemes, to reverse this trend.

## Supplementary Information

Supplementary Material 1.

## Data Availability

The data and code used for meta-analysis are available on GitHub: https://github.com/RuobingMei/Gender-Equity-and-Sustainable-Development_A-Systematic-Review-and-Meta-Analysis.

## References

[1] United Nations. Transforming our world: the 2030 agenda for sustainable development. United Nations; 2015. 25 September.

[2] Gakidou E, Cowling K, Lozano R, Murray CJ. Increased educational attainment and its effect on child mortality in 175 countries between 1970 and 2009: a systematic analysis. Lancet. 2010;376(9745):959–74. 20851260 10.1016/S0140-6736(10)61257-3

[3] Friedman J, York H, Graetz N, Woyczynski L, Whisnant J, Hay SI, et al. Measuring and forecasting progress towards the education-related SDG targets. Nature. 2020;580(7805):636–9. 32350468 10.1038/s41586-020-2198-8 PMC7332421

[4] John S, Singh P. Female education and health: effects of social determinants on economic growth and development. Int J Res Found Hosp Healthc Adm. 2017;5(2):84–8.

[5] Elborgh-Woytek MK, Newiak MM, Kochhar MK, Fabrizio MS, Kpodar MK, Wingender MP, et al. Women, work, and the economy: macroeconomic gains from gender equity. International Monetary Fund; 2013.

[6] Grätz M. The effects of female education on child education: a prospective analysis. Eur Soc. 2023. 10.1080/14616696.2023.2275591.

[7] Augustine J. Increased educational attainment among U.S. mothers and their children’s academic expectations. Res Soc Stratif Mobil. 2017;52:15–25. 29398765 10.1016/j.rssm.2017.08.001 PMC5793933

[8] Balaj M, York HW, Sripada K, Besnier E, Vonen HD, Aravkin A, et al. Parental education and inequalities in child mortality: a global systematic review and meta-analysis. Lancet. 2021;398(10300):608–20. 34119000 10.1016/S0140-6736(21)00534-1 PMC8363948

[9] A life-course. Approach to women’s health. Nat Med. 2024;30(1):1. 38242978 10.1038/s41591-023-02777-8

[10] Hawkes S, Allotey P, Elhadj AS, Clark J, Horton R. The lancet commission on gender and global health. Lancet. 2020;396(10250):521–2. 32763153 10.1016/S0140-6736(20)31547-6 PMC7402649

[11] World Bank. Fertility rate, total (births per woman) 2021 [Available from: https://data.worldbank.org/indicator/SP.DYN.TFRT.IN

[12] Bhattacharjee N, Schumacher A, Aali A, Abate Y, Abbasgholizadeh R, Abbasian M, et al. Global fertility in 204 countries and territories, 1950–2021, with forecasts to 2100: a comprehensive demographic analysis for the Global Burden of Disease Study 2021. Lancet. 2024;403:2057–99. 38521087 10.1016/S0140-6736(24)00550-6 PMC11122687

[13] Vollset SE, Goren E, Yuan CW, Cao J, Smith AE, Hsiao T, et al. Fertility, mortality, migration, and population scenarios for 195 countries and territories from 2017 to 2100: a forecasting analysis for the Global Burden of Disease Study. Lancet. 2020;396(10258):1285–306. 32679112 10.1016/S0140-6736(20)30677-2 PMC7561721

[14] Pacific TLRHW. South korea’s population shift: challenges and opportunities. Lancet Reg Health: Western Pac. 2023;36. 10.1016/j.lanwpc.2023.100865 PMC10447181 37621310

[15] Basten S. Understanding ultra-low fertility in Hong Kong. Low and lower fertility: variations across developed countries. 2015:63–86.

[16] Caltabiano M, Castiglioni M, Rosina A. Lowest-low fertility: signs of a recovery in Italy? Demogr Res. 2009;21:681–718.

[17] Götmark F, Andersson M. Human fertility in relation to education, economy, religion, contraception, and family planning programs. BMC Public Health. 2020;20(1):265. 32087705 10.1186/s12889-020-8331-7 PMC7036237

[18] Kim J. Female education and its impact on fertility. IZA World of Labor; 2023.

[19] Chen J, Guo J. The effect of female education on fertility: evidence from China’s compulsory schooling reform. Econ Educ Rev. 2022;88:102257.

[20] Bongaarts J, editor. The causes of educational differences in fertility in Sub-Saharan Africa2010.

[21] Impicciatore R, Tomatis F. The nexus between education and fertility in six European countries. Genus. 2020;76(1):35.

[22] Boydell V, Mori R, Shahrook S, Gietel-Basten S. Low fertility and fertility policies in the Asia-Pacific region. Global Health Med. 2023;5(5):271–7. 10.35772/ghm.2023.01058 PMC10615026 37908516

[23] Ren Y, Xie Y, Xu Q, Long M, Zheng Y, Li L, et al. University students’ fertility awareness and its influencing factors: a systematic review. Reprod Health. 2023;20(1):85. 37280685 10.1186/s12978-023-01628-6 PMC10242772

[24] Delbaere I, Verbiest S, Tydén T. Knowledge about the impact of age on fertility: a brief review. Ups J Med Sci. 2020;125(2):167–74. 31964217 10.1080/03009734.2019.1707913 PMC7721003

[25] Pedro J, Brandão T, Schmidt L, Costa ME, Martins MV. What do people know about fertility? A systematic review on fertility awareness and its associated factors. Ups J Med Sci. 2018;123(2):71–81. 29957086 10.1080/03009734.2018.1480186 PMC6055749

[26] Page MJ, McKenzie JE, Bossuyt PM, Boutron I, Hoffmann TC, Mulrow CD, et al. The PRISMA 2020 statement: an updated guideline for reporting systematic reviews. BMJ. 2021;372:n71. 33782057 10.1136/bmj.n71 PMC8005924

[27] United Nations. World Population Prospects 2022. United Nations; 2022.

[28] Stang A. Critical evaluation of the Newcastle-Ottawa scale for the assessment of the quality of nonrandomized studies in meta-analyses. Eur J Epidemiol. 2010;25(9):603–5. 20652370 10.1007/s10654-010-9491-z

[29] von Elm E, Altman DG, Egger M, Pocock SJ, Gøtzsche PC, Vandenbroucke JP. Strengthening the reporting of observational studies in epidemiology (STROBE) statement: guidelines for reporting observational studies. BMJ. 2007;335(7624):806–8. 17947786 10.1136/bmj.39335.541782.AD PMC2034723

[30] Lin L, Xu C. Arcsine-based transformations for meta-analysis of proportions: pros, cons, and alternatives. Health Sci Rep. 2020;3(3):e178. 32728636 10.1002/hsr2.178 PMC7384291

[31] Veroniki AA, Jackson D, Viechtbauer W, Bender R, Bowden J, Knapp G, et al. Methods to estimate the between-study variance and its uncertainty in meta-analysis. Res Synth Methods. 2016;7(1):55–79. 26332144 10.1002/jrsm.1164 PMC4950030

[32] Hong QN, Pluye P, Bujold M, Wassef M. Convergent and sequential synthesis designs: implications for conducting and reporting systematic reviews of qualitative and quantitative evidence. Syst Rev. 2017;6(1):61. 28335799 10.1186/s13643-017-0454-2 PMC5364694

[33] Hashemzadeh M, Shariati M, Mohammad Nazari A, Keramat A. Childbearing intention and its associated factors: a systematic review. Nurs Open. 2021;8(5):2354–68. 33705606 10.1002/nop2.849 PMC8363403

[34] Sallis JF, Owen N, Fisher EB. Ecological models of health behavior. In: Health behavior and health education: Theory, research, and practice. 4th ed. San Francisco, CA, US: Jossey-Bass; 2008. p. 465–85.

[35] World Bank. Women, business and the law. Washington, D.C.: World Bank; 2024.

[36] Chan CHY, Chan THY, Peterson BD, Lampic C, Tam MYJ. Intentions and attitudes towards parenthood and fertility awareness among Chinese university students in Hong kong: a comparison with Western samples. Hum Reprod (Oxford England). 2015;30(2):364–72. 10.1093/humrep/deu324 25480921

[37] Lampic C, Svanberg AS, Karlstrom P, Tyden T. Fertility awareness, intentions concerning childbearing, and attitudes towards parenthood among female and male academics. Hum Reprod (Oxford England). 2006;21(2):558–64. 10.1093/humrep/dei367 16293651

[38] Shin H, Lee J, Kim SJ, Jo M. Attitudes towards parenthood and fertility awareness in female and male university students in South Korea. Child Health Nurs Res. 2020;26(3):329–37. 35004476 10.4094/chnr.2020.26.3.329 PMC8650971

[39] Skoog Svanberg A, Lampic C, Karlstrom P-O, Tyden T. Attitudes toward parenthood and awareness of fertility among postgraduate students in Sweden. Gend Med. 2006;3(3):187–95. 17081952 10.1016/s1550-8579(06)80207-x

[40] Sorensen NO, Marcussen S, Backhausen MG, Juhl M, Schmidt L, Tyden T, et al. Fertility awareness and attitudes towards parenthood among Danish university college students. Reproductive Health. 2016;13(1):146. 27964723 10.1186/s12978-016-0258-1 PMC5154162

[41] Tyden T, Svanberg AS, Karlstrom P-O, Lihoff L, Lampic C. Female university students’ attitudes to future motherhood and their understanding about fertility. Eur J Contracept Reproductive Health Care: Official J Eur Soc Contracept. 2006;11(3):181–9. 10.1080/13625180600557803 17056448

[42] Vujcic I, Radicevic T, Dubljanin E, Maksimovic N, Grujicic S. ra. Serbian medical students’ fertility awareness and attitudes towards future parenthood. Eur J Contracept Reproductive Health Care: Official J Eur Soc Contracept. 2017;22(4):291–7. 10.1080/13625187.2017.1368478 28902528

[43] Mogilevkina I, Stern J, Melnik D, Getsko E, Tyden T. Ukrainian medical students’ attitudes to parenthood and knowledge of fertility. Eur J Contracept Reproductive Health Care: Official J Eur Soc Contracept. 2016;21(2):189–94. 10.3109/13625187.2015.1130221 26796521

[44] Rovei V, Gennarelli G, Lantieri T, Casano S, Revelli A, Massobrio M. Family planning, fertility awareness and knowledge about Italian legislation on assisted reproduction among Italian academic students. Reprod Biomed Online. 2010;20(7):873–9. 20418165 10.1016/j.rbmo.2010.03.024

[45] Xu J, Li L, Ma X-Q, Zhang M, Qiao J, Redding SR et al. Fertility Intentions, parenting Attitudes, and fear of childbirth among college students in china: A Cross-Sectional study. J Pediatr Adolesc Gynecol. 2022. 10.1016/j.jpag.2022.07.015 35933080

[46] Safari-Faramani R, Haghdoost AA, Baneshi MR, Dehnavieh R. Exploring the perception of childbearing barriers in a low fertility subgroup of Iran: a qualitative study. Electron Physician. 2018;10(6):6927–34. 30034660 10.19082/6927 PMC6049976

[47] Brinton MC, Bueno X, Oláh L, Hellum M. Postindustrial fertility ideals, intentions, and gender inequality: a comparative qualitative analysis. Popul Dev Rev. 2018;44(2):281–309.

[48] Stack SW, Jagsi R, Biermann JS, Lundberg GP, Law KL, Milne CK, et al. Childbearing decisions in residency: a multicenter survey of female residents. Acad Med. 2020;95(10):1550–7. 32568852 10.1097/ACM.0000000000003549

[49] Grace B, Shawe J, Johnson S, Usman NO, Stephenson J. The ABC of reproductive intentions: a mixed-methods study exploring the spectrum of attitudes towards family building. Hum Reprod. 2022;37(5):988–96. 35238351 10.1093/humrep/deac036 PMC9071225

[50] Spence NJ. Opportunity costs and Latinas’ family formation attitudes. Hispanic J Behav Sci. 2016;38(2):186–205.

[51] Birch Petersen K, Sylvest R, Nyboe Andersen A, Pinborg A, Westring Hvidman H, Schmidt L. Attitudes towards family formation in cohabiting and single childless women in their mid- to late thirties. Hum Fertil (Cambridge England). 2016;19(1):48–55. 10.3109/14647273.2016.1156171 27006139

[52] Brinton MC, Oh E. Babies, work, or both? Highly educated women’s employment and fertility in East Asia. Am J Sociol. 2019;125(1):105–40.

[53] Chen M, Lo CKM, Chen Q, Chan KL, Ip P. Fertility intention in Hong Kong: declining trend and associated factors. Appl Res Qual Life. 2024;19(3):1309–35.

[54] Kim T. The impact of working hours on pregnancy intention in childbearing-age women in Korea, the country with the world’s lowest fertility rate. PLoS One. 2023;18(7):e0288697. 37467184 10.1371/journal.pone.0288697 PMC10355408

[55] Raymo JM, Park H, Xie Y, Yeung WJ. Marriage and family in East Asia: continuity and change. Annu Rev Sociol. 2015;41:471–92. 30078932 10.1146/annurev-soc-073014-112428 PMC6070151

[56] Rosina A, Testa MR. Couples’ first child intentions and disagreement: an analysis of the Italian case. Eur J Popul / Revue européenne De Démographie. 2009;25(4):487–502.

[57] Barker R, Buber-Ennser I. Uncertainty and flexibility of fertility intentions. Adv Life Course Res. 2024;61:100618. 38889542 10.1016/j.alcr.2024.100618

[58] Thomas J, Rowe F, Williamson P, Lin ES. The effect of leave policies on increasing fertility: a systematic review. Humanit Soc Sci Commun. 2022. 10.1057/s41599-022-01270-w.

[59] Bassford M, Fisher H. The impact of paid parental leave on fertility intentions. Econ Rec. 2020;96(315):402–30.

[60] Jung YH, Jang YS, Park E-C. Impact of parental leave system on the childbirth plan among working married women: a three-year follow-up study of the Korean longitudinal survey of women and families. BMC Pregnancy Childbirth. 2024;24(1):99. 38302881 10.1186/s12884-024-06286-5 PMC10832238

[61] Schober PS. Parental leave and domestic work of mothers and fathers: a longitudinal study of two reforms in West Germany. J Social Policy. 2014;43(2):351–72.

[62] Harrison G, Fitzgerald K, O’Leary P, Kothari A, Callaway L. Promoting men-inclusive maternity services: exploring the expectations, experiences and needs of men as fathers. BMC Pregnancy Childbirth. 2024;24(1):477. 38997650 10.1186/s12884-024-06644-3 PMC11245863

[63] Fanelli E, Profeta P. Fathers’ involvement in the family, fertility, and maternal employment: evidence from central and Eastern Europe. Demography. 2021;58(5):1931–54. 34369567 10.1215/00703370-9411306 PMC9807283

[64] Leocádio V, Verona AP, Wajnman S. Exploring the association between gender equality in the family and fertility intentions: an explanation of the findings in low-fertility countries. Genus. 2024;80(1):26.

[65] Clark D. The Capability Approach: Its Development, Critiques and Recent Advances. 2005.

[66] Ferschl S, Gelius P, Abu-Omar K, Till M, Benkert R, Abel T. Exploring well-being and its correlates among young men using Sen’s capability approach: results from the young adults Survey, Switzerland (YASS). Int J Environ Res Public Health. 2022;19(3):1247. 35162270 10.3390/ijerph19031247 PMC8835442

[67] Fraser N. Social justice in the age of identity politics: Redistribution, recognition, and participation. In: Geographic thought. Routledge; 2008. p. 72–89.

[68] Bakkensen JB, Smith KS, Cheung EO, Moreno PI, Goldman KN, Lawson AK, et al. Childbearing, infertility, and career trajectories among women in medicine. JAMA Netw Open. 2023;6(7):e2326192-e. 37498595 10.1001/jamanetworkopen.2023.26192 PMC10375303

[69] Bakkensen JB, Goldman KN. Women’s preventive services initiative: fertility counseling overlooked. Am J Obstet Gynecol. 2022;226(4):524–8. 34228971 10.1016/j.ajog.2021.06.100

[70] Borrowman JD, Unke M, Jones MA, Whitaker KM. A qualitative study describing experiences of pregnancy discrimination in the workplace. J Occup Environ Med. 2024;66(8):e338–42. 38729198 10.1097/JOM.0000000000003136

[71] Wang S, Dong H. Flexible working arrangements and fertility intentions: a survey experiment in Singapore. Eur J Popul. 2024;40(1):33. 39570448 10.1007/s10680-024-09719-1 PMC11582245

[72] Bratsberg B, Walther S. The impact of flexibility at work on fertility. Institute for Fiscal Studies; 2024.

[73] Vitali MJA, Edinburgh. Corporate Welfare, flexible work and fertility intentions. European Population Conference, Scotland: European Association for Population Studies; 2024.

[74] Sobotka T, Matysiak A, Brzozowska Z. Policy responses to low fertility: how effective are they. United Nations Population Fund; 2019.

