# Supplementary material for: Gender equity and sustainable development through the lens of fertility intentions among highly educated women: a systematic review and meta-analysis
Source: BMC Public Health. 2025 Dec 2;25:4427. doi: 10.1186/s12889-025-25842-y (PMC12754999; doi:10.1186/s12889-025-25842-y)
Supplement: Supplementary file 1 — Supplementary Material 1. [file 12889_2025_25842_MOESM1_ESM.docx]

Supplementary Materials

[Tables 2](#_Toc202343110)

[Table S1. Search strategy. 2](#_Toc202343111)

[Table S2. Inclusion and exclusion criteria. 8](#_Toc202343112)

[Table S3. Risk of bias assessment for eligible studies. 9](#_Toc202343113)

[Table S4. Sources of heterogeneity assessed using meta-regression. 10](#_Toc202343114)

[Table S5. Egger’s regression test for funnel plot asymmetry assessment. 10](#_Toc202343115)

[Table S6. Factors associated with fertility intentions in quantitative studies. 11](#_Toc202343116)

[Table S7. Factors associated with fertility intentions in qualitative and mixed-method studies. 15](#_Toc202343117)

[Figures 25](#_Toc202343118)

[Figure S1. Within-region variations in the proportion of highly educated women who intend not to have children. 25](#_Toc202343119)

[Figure S2. Sensitivity analysis using leave-one-out analysis. 26](#_Toc202343120)

[Figure S3. Funnel plot for publication bias assessment. 28](#_Toc202343121)

[Figure S4. Social-ecological model of factors associated with fertility intentions. 29](#_Toc202343122)

[Supplementary References 30](#_Toc202343123)

# Tables

## Table S1. Search strategy.

| **Database** | **Search Strategy** | **Results #** |
| --- | --- | --- |
| **Medline** | (((*Fertility/ or *Reproductive Behavior/) and (exp Intention/ or exp Patient Preference/ or exp Decision Making/ or exp Health Knowledge, Attitudes, Practice/ or exp Social Determinants of Health/ or exp Epidemiologic Factors/)) or ((birth or pregnancy or parenthood or childbirth or child-birth or childbear* or child-bear* or fertility or reproductive or (hav* adj3 child*)) adj3 (intention* or motivation* or opinion* or awareness* or concern* or hesitat* or ambivalen* or preference* or belief* or perspective* or practi#e* or behavior or behaviour or choice* or attitude* or willingness* or ((decision* adj2 mak*) or decision-making))).tw.) and ((women or woman or female* or matern*).tw. or exp Women, Working/) and (exp Educational Status/ or (universit* or college or master* or PhD or postgraduat* or post-graducat* or education* or undergraduat* or under-graduat* or bachelor or gaozhi or gao-zhi).tw.) and ((Saint Kitts and Nevis).mp. or (Serbia or Serb or Serbian).mp. or (Kosovo or Kosovar or Kosovan).mp. or (Norway or Norwegian).mp. or (Hong Kong or HongKong or HongKongness or Hong Kongness).mp. or exp "Republic of Korea"/ or (Korea or Korean).mp. or British Virgin Island*.mp. or (Saint Barthelemy or Saint-Barthelemois).mp. or exp Singapore/ or Singapore*.mp. or exp Macau/ or (Macao or Macau or Macanese or Macaense).mp. or Taiwan/ or (Taiwan or Taiwanese).mp. or exp San Marino/ or (San Marino or Sammarinese).mp. or exp Andorra/ or (Andorra or Andorran).mp. or exp China/ or (China or Chinese or PRC or People* Republic of China).mp. or exp Aruba/ or (Aruba or Aruban).mp. or (Malta or Maltese).mp. or (Malta or Maltese).mp. or (Cayman Island* or Caymanian).mp. or exp Ukraine/ or (Ukraine or Ukrainian).mp. or exp Anguilla/ or (Anguilla or Anguillian).mp. or exp Spain/ or (Spain or Spaniard or Spanish).mp. or exp Italy/ or (Italy or Italian).mp. or exp Japan/ or (Japan or Japanese).mp. or exp Puerto Rico/ or (Puerto Rico or Puerto Rican).mp. or exp Cyprus/ or (Cyprus or Cypriot).mp. or exp Thailand/ or (Thailand or Thai or Siam or Siamese).mp. or exp "Bosnia and Herzegovina"/ or ("Bosnia and Herzegovina" or Bosnian).mp. or exp Jamaica/ or (Jamaica or Jamaican).mp. or exp Portugal/ or (Portugal or Portuguese).mp. or exp Bermuda/ or (Bermuda or Bermudian or Bermudan).mp. or exp Greece/ or (Greece or Greek or Hellenic).mp. or exp "Republic of North Macedonia"/ or (North Macedonia or Macedonian).mp. or exp Bahamas/ or (Bahamas or Bahamian).mp. or exp Finland/ or (Finland or Finn or Finnish or Finnic).mp. or exp Luxembourg/ or (Luxembourg or Luxembourger or Luxembourgish).mp. or exp Albania/ or (Albania or Albanian).mp. or exp Saint Lucia/ or (Saint Lucia or Saint Lucian).mp. or exp Mauritius/ or (Mauritius or Mauritian).mp. or exp Bhutan/ or (Bhutan or Bhutanese).mp. or exp Cuba/ or (Cuba or Cuban).mp. or exp Croatia/ or (Croatia or Croatian or Croat).mp. or (Guernsey or Guern or Gurn or Guernseymen).mp. or exp Guernsey/ or exp Poland/ or (Poland or Pole or Polish).mp. or exp Canada/ or Canada.mp. or Canadian.mp. or exp "Republic of Belarus"/ or Belarus.mp. or Belarusian.mp. or exp Austria/ or Austria.mp. or Austrian.mp. or exp United Arab Emirates/ or United Arab Emirates.mp. or Emirati.mp. or exp New Jersey/ or (Jersey or Jerseyman or Jerseywoman).mp. or exp Russia/ or (Russian Federation or Russia or Russkie or Russian).mp. or exp Uruguay/ or (Uruguay or Uruguayan).mp. or exp Liechtenstein/ or Liechtenstein.mp. or Liechtensteiner.mp. or exp Switzerland/ or Switzerland.mp. or Swiss.mp. or exp Norway/ or Norway.mp. or Norwegian.mp. or exp Kosovo/ or Kosovo.mp. or Kosovar.mp. or Kosovan.mp. or exp Serbia/ or Serbia.mp. or Serb.mp. or Serbian.mp. or (Kitts and Nevis).mp. or exp "Saint Kitts and Nevis"/ or "Saint Kitts and Nevis".mp. or Kittitian.mp. or Nevisian.mp. or exp Germany/ or exp Germany, East/ or exp Germany, West/ or Germany.mp. or German.mp. or exp Costa Rica/ or Costa Rica.mp. or Tico.mp. or Tica.mp. or Costa Rican.mp. or exp Chile/ or Chile.mp. or Chilean.mp. or exp Sint Maarten/ or Sint Maarten.mp. or Saint-Martin.mp. or Saint Martiner.mp. or Saint-Martinois.mp. or Sint Maartener.mp. or (Isle of Man or Manx).mp. or exp "Saint Pierre and Miquelon"/ or "Pierre and Miquelon".mp. or "Saint-Pierre and Miquelon".mp. or "Pierre and Miquelonnais Pierrian".mp. or Saint-Pierrais Miquelonnais Pierrian.mp. or exp United Kingdom/ or exp Northern Ireland/ or exp Wales/ or exp Scotland/ or United Kingdom.mp. or Brit*.mp. or "U.K.".mp. or English.mp. or Scotland.mp. or Scottish.mp. or Wales.mp. or Welsh.mp. or Northern Ireland.mp. or (Montserrat or Montserratian).mp. or exp Slovakia/ or Slovakia.mp. or Slovak.mp. or Slovenky.mp. or Slavic.mp. or exp Armenia/ or Armenia.mp. or Armenian.mp. or exp Hungary/ or Hungary.mp. or Hungarian.mp. or Magyar.mp. or exp Latvia/ or Latvia.mp. or Latvian.mp. or exp Bulgaria/ or Bulgaria.mp. or Bulgarian.mp. or (Antigua and Barbuda).mp. or exp "Antigua and Barbuda"/ or "Antigua and Barbuda".mp. or Antiguan.mp. or Barbudan.mp. or exp Belgium/ or Belgium.mp. or Belgian.mp. or exp Falkland Islands/ or Falkland Island*.mp. or Falklander.mp. or Kelper.mp. or exp Australia/ or Australia.mp. or Australian.mp. or Aussie.mp. or exp Dominica/ or Dominica.mp. or Dominican.mp. or exp Slovenia/ or Slovenia.mp. or Slovene.mp. or Slovenian.mp. or (Trinidad and Tobago).mp. or exp "Trinidad and Tobago"/ or "Trinidad and Tobago".mp. or Trinidadian.mp. or Tobagonian.mp. or Trinis.mp. or Trinbagonian.mp. or exp Lithuania/ or Lithuania.mp. or Lithuanian.mp. or exp Barbados/ or Barbados.mp. or Barbadian.mp. or Bajan.mp. or (Saint Helena or Saint Helenian).mp. or exp Brazil/ or Brazil.mp. or Brazilian.mp. or exp Netherlands/ or Netherlands.mp. or Dutch.mp. or Nederlander.mp. or Holland.mp. or exp Azerbaijan/ or Azerbaijan.mp. or Azerbaijani.mp. or exp Curacao/ or Curacao.mp. or Curacaoan.mp. or (Sint Eustatius and Saba).mp. or (Bonaire or St Eustatius or Statia or Statian or BES Island*).mp. or (Sint Eustatius and Saba).mp. or exp United States/ or United States.mp. or America.mp. or American.mp. or USA.mp. or "U.S.A".mp. or "the US".mp. or "U.S.".mp. or (Turks and Caicos Islands).mp. or ("Turks and Caicos Island*" or "Turks and Caicos Islander*").mp. or exp Estonia/ or Estonia.mp. or Estonia.mp. or exp Sweden/ or Sweden.mp. or Swede.mp. or Swedish.mp. or exp Montenegro/ or Montenegro.mp. or Montenegrin.mp. or exp Iran/ or Iran.mp. or Iranian.mp. or Persian.mp. or exp Polynesia/ or Polynesia.mp. or Polynesian.mp. or exp Czech Republic/ or Czechia.mp. or Czech.mp. or Czechic.mp. or exp Indian Ocean Islands/ or Maldives.mp. or Maldivian.mp. or exp Colombia/ or Colombia.mp. or Colombian.mp. or exp Denmark/ or Denmark.mp. or Danes.mp. or Danish.mp. or exp Iceland/ or Iceland.mp. or Icelander.mp. or Icelandic.mp. or exp Romania/ or Romania.mp. or Romanian.mp. or Rumanian.mp. or Roumanian.mp. or exp Ireland/ or Ireland.mp. or Irish.mp. or Irelander.mp. or Irishman.mp. or exp Brunei/ or Brunei.mp. or Bruneian.mp. or exp New Zealand/ or New Zealand.mp. or New Zealander.mp. or exp France/ or France.mp. or French.mp. or Frenchman.mp. or Frenchwoman.mp. or (Vincent and the Grenadines).mp. or exp "Saint Vincent and the Grenadines"/ or "Saint Vincent and the Grenadines".mp. or Vincentian.mp. or "Vincent and the Grenadines".mp. or exp Moldova/ or Moldova.mp. or Moldovan.mp. or exp Qatar/ or Qatar.mp. or Qatari.mp. or exp El Salvador/ or El Salvador.mp. or Salvadoran.mp. or Salvadorian.mp. or exp Bahrain/ or Bahrain.mp. or Bahrani.mp. or exp "Democratic People's Republic of Korea"/ or North Korea.mp. or People's Republic of Korea.mp. or North Korean.mp. or Korean.mp. or DPRK.mp. or exp Mexico/ or Mexico.mp. or Mexican.mp. or exp Gibraltar/ or Gibraltar.mp. or Gibraltarian.mp. or exp Argentina/ or Argentina.mp. or Argentinian.mp. or Argentine.mp. or exp Turkey/ or Turkey.mp. or Turkish.mp. or exp Martinique/ or Martinique.mp. or Martiniquais.mp. or Martinican.mp. or exp Cabo Verde/ or Cape Verdean.mp. or Cabo Verdean.mp. or (Wallis and Futuna Islands).mp. or exp "Wallis and Futuna Islands"/ or Wallisian.mp. or Futunan.mp. or "Wallis and Futuna".mp. or (Wallis and Futuna Islands).mp. or exp Vietnam/ or VietNam.mp. or Vietnam.mp. or Vietnamese.mp. or Viet.mp. or exp Greenland/ or Greenland.mp. or Greenlandic.mp. or exp Sri Lanka/ or Sri Lanka.mp. or Sinhalese.mp. or Sri Lankan.mp. or exp Bangladesh/ or Bangladesh.mp. or Bangladeshi.mp. or Bengali.mp. or exp Grenada/ or Grenada.mp. or Grenadian.mp. or exp Belize/ or Belize.mp. or Belizean.mp. or exp New Caledonia/ or New Caledonia.mp. or Kanak.mp. or Kanaky.mp. or exp Ecuador/ or Ecuador.mp. or Ecuadorian.mp. or exp India/ or India.mp. or Indian.mp. or Hindu.mp. or exp Nepal/ or Nepal.mp. or Nepali.mp. or Nepalese.mp. or exp Kuwait/ or Kuwait.mp. or Kuwaiti.mp. or exp Guadeloupe/ or Guadeloupe.mp. or Guadeloupean.mp. or exp "Georgia (Republic)"/ or Georgia.mp. or Georgian.mp. or exp Lebanon/ or Lebanon.mp. or Lebanese.mp. or exp Tunisia/ or Tunisia.mp. or Tunisian.mp. or exp Asians/ or exp Europe/ or exp Europe, Eastern/ or exp European Union/ or exp Asia/ or exp Asia, Southeastern/ or exp Asia, Central/ or exp Asia, Western/ or exp Asia, Northern/ or exp Americas/ or exp Caribbean Region/ or exp Central America/ or exp Latin America/ or exp North America/ or exp South America/)  **Restricted to paper published between 2000 and 2022** | 1917 |
| **Embase** | (((*Fertility/ or *Reproductive Behavior/) and (exp Intention/ or exp Patient Preference/ or exp Decision Making/ or exp Health Knowledge, Attitudes, Practice/ or exp Social Determinants of Health/ or exp Epidemiologic Factors/)) or ((birth or pregnancy or parenthood or childbirth or child-birth or childbear* or child-bear* or fertility or reproductive or (hav* adj3 child*)) adj3 (intention* or motivation* or opinion* or awareness* or concern* or hesitat* or ambivalen* or preference* or belief* or perspective* or practi#e* or behavior or behaviour or choice* or attitude* or willingness* or ((decision* adj2 mak*) or decision-making))).tw.) and ((women or woman or female* or matern*).tw. or exp Women, Working/) and (exp Educational Status/ or (universit* or college or master* or PhD or postgraduat* or post-graducat* or education* or undergraduat* or under-graduat* or bachelor or gaozhi or gao-zhi).tw.) and ((Saint Kitts and Nevis).mp. or (Serbia or Serb or Serbian).mp. or (Kosovo or Kosovar or Kosovan).mp. or (Norway or Norwegian).mp. or (Hong Kong or HongKong or HongKongness or Hong Kongness).mp. or exp "Republic of Korea"/ or (Korea or Korean).mp. or British Virgin Island*.mp. or (Saint Barthelemy or Saint-Barthelemois).mp. or exp Singapore/ or Singapore*.mp. or exp Macau/ or (Macao or Macau or Macanese or Macaense).mp. or Taiwan/ or (Taiwan or Taiwanese).mp. or exp San Marino/ or (San Marino or Sammarinese).mp. or exp Andorra/ or (Andorra or Andorran).mp. or exp China/ or (China or Chinese or PRC or People* Republic of China).mp. or exp Aruba/ or (Aruba or Aruban).mp. or (Malta or Maltese).mp. or (Malta or Maltese).mp. or (Cayman Island* or Caymanian).mp. or exp Ukraine/ or (Ukraine or Ukrainian).mp. or exp Anguilla/ or (Anguilla or Anguillian).mp. or exp Spain/ or (Spain or Spaniard or Spanish).mp. or exp Italy/ or (Italy or Italian).mp. or exp Japan/ or (Japan or Japanese).mp. or exp Puerto Rico/ or (Puerto Rico or Puerto Rican).mp. or exp Cyprus/ or (Cyprus or Cypriot).mp. or exp Thailand/ or (Thailand or Thai or Siam or Siamese).mp. or exp "Bosnia and Herzegovina"/ or ("Bosnia and Herzegovina" or Bosnian).mp. or exp Jamaica/ or (Jamaica or Jamaican).mp. or exp Portugal/ or (Portugal or Portuguese).mp. or exp Bermuda/ or (Bermuda or Bermudian or Bermudan).mp. or exp Greece/ or (Greece or Greek or Hellenic).mp. or exp "Republic of North Macedonia"/ or (North Macedonia or Macedonian).mp. or exp Bahamas/ or (Bahamas or Bahamian).mp. or exp Finland/ or (Finland or Finn or Finnish or Finnic).mp. or exp Luxembourg/ or (Luxembourg or Luxembourger or Luxembourgish).mp. or exp Albania/ or (Albania or Albanian).mp. or exp Saint Lucia/ or (Saint Lucia or Saint Lucian).mp. or exp Mauritius/ or (Mauritius or Mauritian).mp. or exp Bhutan/ or (Bhutan or Bhutanese).mp. or exp Cuba/ or (Cuba or Cuban).mp. or exp Croatia/ or (Croatia or Croatian or Croat).mp. or (Guernsey or Guern or Gurn or Guernseymen).mp. or exp Guernsey/ or exp Poland/ or (Poland or Pole or Polish).mp. or exp Canada/ or Canada.mp. or Canadian.mp. or exp "Republic of Belarus"/ or Belarus.mp. or Belarusian.mp. or exp Austria/ or Austria.mp. or Austrian.mp. or exp United Arab Emirates/ or United Arab Emirates.mp. or Emirati.mp. or exp New Jersey/ or (Jersey or Jerseyman or Jerseywoman).mp. or exp Russia/ or (Russian Federation or Russia or Russkie or Russian).mp. or exp Uruguay/ or (Uruguay or Uruguayan).mp. or exp Liechtenstein/ or Liechtenstein.mp. or Liechtensteiner.mp. or exp Switzerland/ or Switzerland.mp. or Swiss.mp. or exp Norway/ or Norway.mp. or Norwegian.mp. or exp Kosovo/ or Kosovo.mp. or Kosovar.mp. or Kosovan.mp. or exp Serbia/ or Serbia.mp. or Serb.mp. or Serbian.mp. or (Kitts and Nevis).mp. or exp "Saint Kitts and Nevis"/ or "Saint Kitts and Nevis".mp. or Kittitian.mp. or Nevisian.mp. or exp Germany/ or exp Germany, East/ or exp Germany, West/ or Germany.mp. or German.mp. or exp Costa Rica/ or Costa Rica.mp. or Tico.mp. or Tica.mp. or Costa Rican.mp. or exp Chile/ or Chile.mp. or Chilean.mp. or exp Sint Maarten/ or Sint Maarten.mp. or Saint-Martin.mp. or Saint Martiner.mp. or Saint-Martinois.mp. or Sint Maartener.mp. or (Isle of Man or Manx).mp. or exp "Saint Pierre and Miquelon"/ or "Pierre and Miquelon".mp. or "Saint-Pierre and Miquelon".mp. or "Pierre and Miquelonnais Pierrian".mp. or Saint-Pierrais Miquelonnais Pierrian.mp. or exp United Kingdom/ or exp Northern Ireland/ or exp Wales/ or exp Scotland/ or United Kingdom.mp. or Brit*.mp. or "U.K.".mp. or English.mp. or Scotland.mp. or Scottish.mp. or Wales.mp. or Welsh.mp. or Northern Ireland.mp. or (Montserrat or Montserratian).mp. or exp Slovakia/ or Slovakia.mp. or Slovak.mp. or Slovenky.mp. or Slavic.mp. or exp Armenia/ or Armenia.mp. or Armenian.mp. or exp Hungary/ or Hungary.mp. or Hungarian.mp. or Magyar.mp. or exp Latvia/ or Latvia.mp. or Latvian.mp. or exp Bulgaria/ or Bulgaria.mp. or Bulgarian.mp. or (Antigua and Barbuda).mp. or exp "Antigua and Barbuda"/ or "Antigua and Barbuda".mp. or Antiguan.mp. or Barbudan.mp. or exp Belgium/ or Belgium.mp. or Belgian.mp. or exp Falkland Islands/ or Falkland Island*.mp. or Falklander.mp. or Kelper.mp. or exp Australia/ or Australia.mp. or Australian.mp. or Aussie.mp. or exp Dominica/ or Dominica.mp. or Dominican.mp. or exp Slovenia/ or Slovenia.mp. or Slovene.mp. or Slovenian.mp. or (Trinidad and Tobago).mp. or exp "Trinidad and Tobago"/ or "Trinidad and Tobago".mp. or Trinidadian.mp. or Tobagonian.mp. or Trinis.mp. or Trinbagonian.mp. or exp Lithuania/ or Lithuania.mp. or Lithuanian.mp. or exp Barbados/ or Barbados.mp. or Barbadian.mp. or Bajan.mp. or (Saint Helena or Saint Helenian).mp. or exp Brazil/ or Brazil.mp. or Brazilian.mp. or exp Netherlands/ or Netherlands.mp. or Dutch.mp. or Nederlander.mp. or Holland.mp. or exp Azerbaijan/ or Azerbaijan.mp. or Azerbaijani.mp. or exp Curacao/ or Curacao.mp. or Curacaoan.mp. or (Sint Eustatius and Saba).mp. or (Bonaire or St Eustatius or Statia or Statian or BES Island*).mp. or (Sint Eustatius and Saba).mp. or exp United States/ or United States.mp. or America.mp. or American.mp. or USA.mp. or "U.S.A".mp. or "the US".mp. or "U.S.".mp. or (Turks and Caicos Islands).mp. or ("Turks and Caicos Island*" or "Turks and Caicos Islander*").mp. or exp Estonia/ or Estonia.mp. or Estonia.mp. or exp Sweden/ or Sweden.mp. or Swede.mp. or Swedish.mp. or exp Montenegro/ or Montenegro.mp. or Montenegrin.mp. or exp Iran/ or Iran.mp. or Iranian.mp. or Persian.mp. or exp Polynesia/ or Polynesia.mp. or Polynesian.mp. or exp Czech Republic/ or Czechia.mp. or Czech.mp. or Czechic.mp. or exp Indian Ocean Islands/ or Maldives.mp. or Maldivian.mp. or exp Colombia/ or Colombia.mp. or Colombian.mp. or exp Denmark/ or Denmark.mp. or Danes.mp. or Danish.mp. or exp Iceland/ or Iceland.mp. or Icelander.mp. or Icelandic.mp. or exp Romania/ or Romania.mp. or Romanian.mp. or Rumanian.mp. or Roumanian.mp. or exp Ireland/ or Ireland.mp. or Irish.mp. or Irelander.mp. or Irishman.mp. or exp Brunei/ or Brunei.mp. or Bruneian.mp. or exp New Zealand/ or New Zealand.mp. or New Zealander.mp. or exp France/ or France.mp. or French.mp. or Frenchman.mp. or Frenchwoman.mp. or (Vincent and the Grenadines).mp. or exp "Saint Vincent and the Grenadines"/ or "Saint Vincent and the Grenadines".mp. or Vincentian.mp. or "Vincent and the Grenadines".mp. or exp Moldova/ or Moldova.mp. or Moldovan.mp. or exp Qatar/ or Qatar.mp. or Qatari.mp. or exp El Salvador/ or El Salvador.mp. or Salvadoran.mp. or Salvadorian.mp. or exp Bahrain/ or Bahrain.mp. or Bahrani.mp. or exp "Democratic People's Republic of Korea"/ or North Korea.mp. or People's Republic of Korea.mp. or North Korean.mp. or Korean.mp. or DPRK.mp. or exp Mexico/ or Mexico.mp. or Mexican.mp. or exp Gibraltar/ or Gibraltar.mp. or Gibraltarian.mp. or exp Argentina/ or Argentina.mp. or Argentinian.mp. or Argentine.mp. or exp Turkey/ or Turkey.mp. or Turkish.mp. or exp Martinique/ or Martinique.mp. or Martiniquais.mp. or Martinican.mp. or exp Cabo Verde/ or Cape Verdean.mp. or Cabo Verdean.mp. or (Wallis and Futuna Islands).mp. or exp "Wallis and Futuna Islands"/ or Wallisian.mp. or Futunan.mp. or "Wallis and Futuna".mp. or (Wallis and Futuna Islands).mp. or exp Vietnam/ or VietNam.mp. or Vietnam.mp. or Vietnamese.mp. or Viet.mp. or exp Greenland/ or Greenland.mp. or Greenlandic.mp. or exp Sri Lanka/ or Sri Lanka.mp. or Sinhalese.mp. or Sri Lankan.mp. or exp Bangladesh/ or Bangladesh.mp. or Bangladeshi.mp. or Bengali.mp. or exp Grenada/ or Grenada.mp. or Grenadian.mp. or exp Belize/ or Belize.mp. or Belizean.mp. or exp New Caledonia/ or New Caledonia.mp. or Kanak.mp. or Kanaky.mp. or exp Ecuador/ or Ecuador.mp. or Ecuadorian.mp. or exp India/ or India.mp. or Indian.mp. or Hindu.mp. or exp Nepal/ or Nepal.mp. or Nepali.mp. or Nepalese.mp. or exp Kuwait/ or Kuwait.mp. or Kuwaiti.mp. or exp Guadeloupe/ or Guadeloupe.mp. or Guadeloupean.mp. or exp "Georgia (Republic)"/ or Georgia.mp. or Georgian.mp. or exp Lebanon/ or Lebanon.mp. or Lebanese.mp. or exp Tunisia/ or Tunisia.mp. or Tunisian.mp. or exp Asians/ or exp Europe/ or exp Europe, Eastern/ or exp European Union/ or exp Asia/ or exp Asia, Southeastern/ or exp Asia, Central/ or exp Asia, Western/ or exp Asia, Northern/ or exp Americas/ or exp Caribbean Region/ or exp Central America/ or exp Latin America/ or exp North America/ or exp South America/)  **Restricted to articles published between 2000 and 2022 and removed conference abstracts** | 1912 |
| **Global Health** | (((birth or pregnancy or parenthood or childbirth or child-birth or childbear* or child-bear* or fertility or reproductive or (hav* adj3 child*)) adj3 (intention* or motivation* or opinion* or awareness* or concern* or hesitat* or ambivalen* or preference* or belief* or perspective* or practi#e* or behavior or behaviour or choice* or attitude* or willingness* or ((decision* adj2 mak*) or decision-making))).tw. or ((female fertility/ or human fertility/ or fertility/ or reproductive behaviour/) and ((attitudes or behaviour or human behaviour).sh. or exp practice/ or exp decision making/ or health determinants/ or risk factors.sh.))) and ((women or woman or female* or matern*).tw. or exp Women, Working/) and (exp Educational Status/ or (universit* or college or master* or PhD or postgraduat* or post-graducat* or education* or undergraduat* or under-graduat* or bachelor or gaozhi or gao-zhi).tw. or exp education/ or exp adult education/) and ((Saint Kitts and Nevis).mp. or (Serbia or Serb or Serbian).mp. or (Kosovo or Kosovar or Kosovan).mp. or (Norway or Norwegian).mp. or (Hong Kong or HongKong or HongKongness or Hong Kongness).mp. or exp "Republic of Korea"/ or (Korea or Korean).mp. or British Virgin Island*.mp. or (Saint Barthelemy or Saint-Barthelemois).mp. or exp Singapore/ or Singapore*.mp. or exp Macau/ or (Macao or Macau or Macanese or Macaense).mp. or Taiwan/ or (Taiwan or Taiwanese).mp. or exp San Marino/ or (San Marino or Sammarinese).mp. or exp Andorra/ or (Andorra or Andorran).mp. or exp China/ or (China or Chinese or PRC or People* Republic of China).mp. or exp Aruba/ or (Aruba or Aruban).mp. or (Malta or Maltese).mp. or (Malta or Maltese).mp. or (Cayman Island* or Caymanian).mp. or exp Ukraine/ or (Ukraine or Ukrainian).mp. or exp Anguilla/ or (Anguilla or Anguillian).mp. or exp Spain/ or (Spain or Spaniard or Spanish).mp. or exp Italy/ or (Italy or Italian).mp. or exp Japan/ or (Japan or Japanese).mp. or exp Puerto Rico/ or (Puerto Rico or Puerto Rican).mp. or exp Cyprus/ or (Cyprus or Cypriot).mp. or exp Thailand/ or (Thailand or Thai or Siam or Siamese).mp. or exp "Bosnia and Herzegovina"/ or ("Bosnia and Herzegovina" or Bosnian).mp. or exp Jamaica/ or (Jamaica or Jamaican).mp. or exp Portugal/ or (Portugal or Portuguese).mp. or exp Bermuda/ or (Bermuda or Bermudian or Bermudan).mp. or exp Greece/ or (Greece or Greek or Hellenic).mp. or exp "Republic of North Macedonia"/ or (North Macedonia or Macedonian).mp. or exp Bahamas/ or (Bahamas or Bahamian).mp. or exp Finland/ or (Finland or Finn or Finnish or Finnic).mp. or exp Luxembourg/ or (Luxembourg or Luxembourger or Luxembourgish).mp. or exp Albania/ or (Albania or Albanian).mp. or exp Saint Lucia/ or (Saint Lucia or Saint Lucian).mp. or exp Mauritius/ or (Mauritius or Mauritian).mp. or exp Bhutan/ or (Bhutan or Bhutanese).mp. or exp Cuba/ or (Cuba or Cuban).mp. or exp Croatia/ or (Croatia or Croatian or Croat).mp. or (Guernsey or Guern or Gurn or Guernseymen).mp. or exp Guernsey/ or exp Poland/ or (Poland or Pole or Polish).mp. or exp Canada/ or Canada.mp. or Canadian.mp. or exp "Republic of Belarus"/ or Belarus.mp. or Belarusian.mp. or exp Austria/ or Austria.mp. or Austrian.mp. or exp United Arab Emirates/ or United Arab Emirates.mp. or Emirati.mp. or exp New Jersey/ or (Jersey or Jerseyman or Jerseywoman).mp. or exp Russia/ or (Russian Federation or Russia or Russkie or Russian).mp. or exp Uruguay/ or (Uruguay or Uruguayan).mp. or exp Liechtenstein/ or Liechtenstein.mp. or Liechtensteiner.mp. or exp Switzerland/ or Switzerland.mp. or Swiss.mp. or exp Norway/ or Norway.mp. or Norwegian.mp. or exp Kosovo/ or Kosovo.mp. or Kosovar.mp. or Kosovan.mp. or exp Serbia/ or Serbia.mp. or Serb.mp. or Serbian.mp. or (Kitts and Nevis).mp. or exp "Saint Kitts and Nevis"/ or "Saint Kitts and Nevis".mp. or Kittitian.mp. or Nevisian.mp. or exp Germany/ or exp Germany, East/ or exp Germany, West/ or Germany.mp. or German.mp. or exp Costa Rica/ or Costa Rica.mp. or Tico.mp. or Tica.mp. or Costa Rican.mp. or exp Chile/ or Chile.mp. or Chilean.mp. or exp Sint Maarten/ or Sint Maarten.mp. or Saint-Martin.mp. or Saint Martiner.mp. or Saint-Martinois.mp. or Sint Maartener.mp. or (Isle of Man or Manx).mp. or exp "Saint Pierre and Miquelon"/ or "Pierre and Miquelon".mp. or "Saint-Pierre and Miquelon".mp. or "Pierre and Miquelonnais Pierrian".mp. or Saint-Pierrais Miquelonnais Pierrian.mp. or exp United Kingdom/ or exp Northern Ireland/ or exp Wales/ or exp Scotland/ or United Kingdom.mp. or Brit*.mp. or "U.K.".mp. or English.mp. or Scotland.mp. or Scottish.mp. or Wales.mp. or Welsh.mp. or Northern Ireland.mp. or (Montserrat or Montserratian).mp. or exp Slovakia/ or Slovakia.mp. or Slovak.mp. or Slovenky.mp. or Slavic.mp. or exp Armenia/ or Armenia.mp. or Armenian.mp. or exp Hungary/ or Hungary.mp. or Hungarian.mp. or Magyar.mp. or exp Latvia/ or Latvia.mp. or Latvian.mp. or exp Bulgaria/ or Bulgaria.mp. or Bulgarian.mp. or (Antigua and Barbuda).mp. or exp "Antigua and Barbuda"/ or "Antigua and Barbuda".mp. or Antiguan.mp. or Barbudan.mp. or exp Belgium/ or Belgium.mp. or Belgian.mp. or exp Falkland Islands/ or Falkland Island*.mp. or Falklander.mp. or Kelper.mp. or exp Australia/ or Australia.mp. or Australian.mp. or Aussie.mp. or exp Dominica/ or Dominica.mp. or Dominican.mp. or exp Slovenia/ or Slovenia.mp. or Slovene.mp. or Slovenian.mp. or (Trinidad and Tobago).mp. or exp "Trinidad and Tobago"/ or "Trinidad and Tobago".mp. or Trinidadian.mp. or Tobagonian.mp. or Trinis.mp. or Trinbagonian.mp. or exp Lithuania/ or Lithuania.mp. or Lithuanian.mp. or exp Barbados/ or Barbados.mp. or Barbadian.mp. or Bajan.mp. or (Saint Helena or Saint Helenian).mp. or exp Brazil/ or Brazil.mp. or Brazilian.mp. or exp Netherlands/ or Netherlands.mp. or Dutch.mp. or Nederlander.mp. or Holland.mp. or exp Azerbaijan/ or Azerbaijan.mp. or Azerbaijani.mp. or exp Curacao/ or Curacao.mp. or Curacaoan.mp. or (Sint Eustatius and Saba).mp. or (Bonaire or St Eustatius or Statia or Statian or BES Island*).mp. or (Sint Eustatius and Saba).mp. or exp United States/ or United States.mp. or America.mp. or American.mp. or USA.mp. or "U.S.A".mp. or "the US".mp. or "U.S.".mp. or (Turks and Caicos Islands).mp. or ("Turks and Caicos Island*" or "Turks and Caicos Islander*").mp. or exp Estonia/ or Estonia.mp. or Estonia.mp. or exp Sweden/ or Sweden.mp. or Swede.mp. or Swedish.mp. or exp Montenegro/ or Montenegro.mp. or Montenegrin.mp. or exp Iran/ or Iran.mp. or Iranian.mp. or Persian.mp. or exp Polynesia/ or Polynesia.mp. or Polynesian.mp. or exp Czech Republic/ or Czechia.mp. or Czech.mp. or Czechic.mp. or exp Indian Ocean Islands/ or Maldives.mp. or Maldivian.mp. or exp Colombia/ or Colombia.mp. or Colombian.mp. or exp Denmark/ or Denmark.mp. or Danes.mp. or Danish.mp. or exp Iceland/ or Iceland.mp. or Icelander.mp. or Icelandic.mp. or exp Romania/ or Romania.mp. or Romanian.mp. or Rumanian.mp. or Roumanian.mp. or exp Ireland/ or Ireland.mp. or Irish.mp. or Irelander.mp. or Irishman.mp. or exp Brunei/ or Brunei.mp. or Bruneian.mp. or exp New Zealand/ or New Zealand.mp. or New Zealander.mp. or exp France/ or France.mp. or French.mp. or Frenchman.mp. or Frenchwoman.mp. or (Vincent and the Grenadines).mp. or exp "Saint Vincent and the Grenadines"/ or "Saint Vincent and the Grenadines".mp. or Vincentian.mp. or "Vincent and the Grenadines".mp. or exp Moldova/ or Moldova.mp. or Moldovan.mp. or exp Qatar/ or Qatar.mp. or Qatari.mp. or exp El Salvador/ or El Salvador.mp. or Salvadoran.mp. or Salvadorian.mp. or exp Bahrain/ or Bahrain.mp. or Bahrani.mp. or exp "Democratic People's Republic of Korea"/ or North Korea.mp. or People's Republic of Korea.mp. or North Korean.mp. or Korean.mp. or DPRK.mp. or exp Mexico/ or Mexico.mp. or Mexican.mp. or exp Gibraltar/ or Gibraltar.mp. or Gibraltarian.mp. or exp Argentina/ or Argentina.mp. or Argentinian.mp. or Argentine.mp. or exp Turkey/ or Turkey.mp. or Turkish.mp. or exp Martinique/ or Martinique.mp. or Martiniquais.mp. or Martinican.mp. or exp Cabo Verde/ or Cape Verdean.mp. or Cabo Verdean.mp. or (Wallis and Futuna Islands).mp. or exp "Wallis and Futuna Islands"/ or Wallisian.mp. or Futunan.mp. or "Wallis and Futuna".mp. or (Wallis and Futuna Islands).mp. or exp Vietnam/ or VietNam.mp. or Vietnam.mp. or Vietnamese.mp. or Viet.mp. or exp Greenland/ or Greenland.mp. or Greenlandic.mp. or exp Sri Lanka/ or Sri Lanka.mp. or Sinhalese.mp. or Sri Lankan.mp. or exp Bangladesh/ or Bangladesh.mp. or Bangladeshi.mp. or Bengali.mp. or exp Grenada/ or Grenada.mp. or Grenadian.mp. or exp Belize/ or Belize.mp. or Belizean.mp. or exp New Caledonia/ or New Caledonia.mp. or Kanak.mp. or Kanaky.mp. or exp Ecuador/ or Ecuador.mp. or Ecuadorian.mp. or exp India/ or India.mp. or Indian.mp. or Hindu.mp. or exp Nepal/ or Nepal.mp. or Nepali.mp. or Nepalese.mp. or exp Kuwait/ or Kuwait.mp. or Kuwaiti.mp. or exp Guadeloupe/ or Guadeloupe.mp. or Guadeloupean.mp. or exp "Georgia (Republic)"/ or Georgia.mp. or Georgian.mp. or exp Lebanon/ or Lebanon.mp. or Lebanese.mp. or exp Tunisia/ or Tunisia.mp. or Tunisian.mp. or exp Asians/ or exp Europe/ or exp Europe, Eastern/ or exp European Union/ or exp Asia/ or exp Asia, Southeastern/ or exp Asia, Central/ or exp Asia, Western/ or exp Asia, Northern/ or exp Americas/ or exp Caribbean Region/ or exp Central America/ or exp Latin America/ or exp North America/ or exp South America/)  **Restricted to articles published between 2000 and 2022 and removed conference proceedings** | 970 |
| **APA PsycExtra** | (((*Fertility/ or *Reproductive Behavior/) and (exp Intention/ or exp Patient Preference/ or exp Decision Making/ or exp Health Knowledge, Attitudes, Practice/ or exp Social Determinants of Health/ or exp Epidemiologic Factors/)) or ((birth or pregnancy or parenthood or childbirth or child-birth or childbear* or child-bear* or fertility or reproductive or (hav* adj3 child*)) adj3 (intention* or motivation* or opinion* or awareness* or concern* or hesitat* or ambivalen* or preference* or belief* or perspective* or practi#e* or behavior or behaviour or choice* or attitude* or willingness* or ((decision* adj2 mak*) or decision-making))).tw.) and ((women or woman or female* or matern*).tw. or exp Women, Working/) and (exp Educational Status/ or (universit* or college or master* or PhD or postgraduat* or post-graducat* or education* or undergraduat* or under-graduat* or bachelor or gaozhi or gao-zhi).tw.) and ((Saint Kitts and Nevis).mp. or (Serbia or Serb or Serbian).mp. or (Kosovo or Kosovar or Kosovan).mp. or (Norway or Norwegian).mp. or (Hong Kong or HongKong or HongKongness or Hong Kongness).mp. or exp "Republic of Korea"/ or (Korea or Korean).mp. or British Virgin Island*.mp. or (Saint Barthelemy or Saint-Barthelemois).mp. or exp Singapore/ or Singapore*.mp. or exp Macau/ or (Macao or Macau or Macanese or Macaense).mp. or Taiwan/ or (Taiwan or Taiwanese).mp. or exp San Marino/ or (San Marino or Sammarinese).mp. or exp Andorra/ or (Andorra or Andorran).mp. or exp China/ or (China or Chinese or PRC or People* Republic of China).mp. or exp Aruba/ or (Aruba or Aruban).mp. or (Malta or Maltese).mp. or (Malta or Maltese).mp. or (Cayman Island* or Caymanian).mp. or exp Ukraine/ or (Ukraine or Ukrainian).mp. or exp Anguilla/ or (Anguilla or Anguillian).mp. or exp Spain/ or (Spain or Spaniard or Spanish).mp. or exp Italy/ or (Italy or Italian).mp. or exp Japan/ or (Japan or Japanese).mp. or exp Puerto Rico/ or (Puerto Rico or Puerto Rican).mp. or exp Cyprus/ or (Cyprus or Cypriot).mp. or exp Thailand/ or (Thailand or Thai or Siam or Siamese).mp. or exp "Bosnia and Herzegovina"/ or ("Bosnia and Herzegovina" or Bosnian).mp. or exp Jamaica/ or (Jamaica or Jamaican).mp. or exp Portugal/ or (Portugal or Portuguese).mp. or exp Bermuda/ or (Bermuda or Bermudian or Bermudan).mp. or exp Greece/ or (Greece or Greek or Hellenic).mp. or exp "Republic of North Macedonia"/ or (North Macedonia or Macedonian).mp. or exp Bahamas/ or (Bahamas or Bahamian).mp. or exp Finland/ or (Finland or Finn or Finnish or Finnic).mp. or exp Luxembourg/ or (Luxembourg or Luxembourger or Luxembourgish).mp. or exp Albania/ or (Albania or Albanian).mp. or exp Saint Lucia/ or (Saint Lucia or Saint Lucian).mp. or exp Mauritius/ or (Mauritius or Mauritian).mp. or exp Bhutan/ or (Bhutan or Bhutanese).mp. or exp Cuba/ or (Cuba or Cuban).mp. or exp Croatia/ or (Croatia or Croatian or Croat).mp. or (Guernsey or Guern or Gurn or Guernseymen).mp. or exp Guernsey/ or exp Poland/ or (Poland or Pole or Polish).mp. or exp Canada/ or Canada.mp. or Canadian.mp. or exp "Republic of Belarus"/ or Belarus.mp. or Belarusian.mp. or exp Austria/ or Austria.mp. or Austrian.mp. or exp United Arab Emirates/ or United Arab Emirates.mp. or Emirati.mp. or exp New Jersey/ or (Jersey or Jerseyman or Jerseywoman).mp. or exp Russia/ or (Russian Federation or Russia or Russkie or Russian).mp. or exp Uruguay/ or (Uruguay or Uruguayan).mp. or exp Liechtenstein/ or Liechtenstein.mp. or Liechtensteiner.mp. or exp Switzerland/ or Switzerland.mp. or Swiss.mp. or exp Norway/ or Norway.mp. or Norwegian.mp. or exp Kosovo/ or Kosovo.mp. or Kosovar.mp. or Kosovan.mp. or exp Serbia/ or Serbia.mp. or Serb.mp. or Serbian.mp. or (Kitts and Nevis).mp. or exp "Saint Kitts and Nevis"/ or "Saint Kitts and Nevis".mp. or Kittitian.mp. or Nevisian.mp. or exp Germany/ or exp Germany, East/ or exp Germany, West/ or Germany.mp. or German.mp. or exp Costa Rica/ or Costa Rica.mp. or Tico.mp. or Tica.mp. or Costa Rican.mp. or exp Chile/ or Chile.mp. or Chilean.mp. or exp Sint Maarten/ or Sint Maarten.mp. or Saint-Martin.mp. or Saint Martiner.mp. or Saint-Martinois.mp. or Sint Maartener.mp. or (Isle of Man or Manx).mp. or exp "Saint Pierre and Miquelon"/ or "Pierre and Miquelon".mp. or "Saint-Pierre and Miquelon".mp. or "Pierre and Miquelonnais Pierrian".mp. or Saint-Pierrais Miquelonnais Pierrian.mp. or exp United Kingdom/ or exp Northern Ireland/ or exp Wales/ or exp Scotland/ or United Kingdom.mp. or Brit*.mp. or "U.K.".mp. or English.mp. or Scotland.mp. or Scottish.mp. or Wales.mp. or Welsh.mp. or Northern Ireland.mp. or (Montserrat or Montserratian).mp. or exp Slovakia/ or Slovakia.mp. or Slovak.mp. or Slovenky.mp. or Slavic.mp. or exp Armenia/ or Armenia.mp. or Armenian.mp. or exp Hungary/ or Hungary.mp. or Hungarian.mp. or Magyar.mp. or exp Latvia/ or Latvia.mp. or Latvian.mp. or exp Bulgaria/ or Bulgaria.mp. or Bulgarian.mp. or (Antigua and Barbuda).mp. or exp "Antigua and Barbuda"/ or "Antigua and Barbuda".mp. or Antiguan.mp. or Barbudan.mp. or exp Belgium/ or Belgium.mp. or Belgian.mp. or exp Falkland Islands/ or Falkland Island*.mp. or Falklander.mp. or Kelper.mp. or exp Australia/ or Australia.mp. or Australian.mp. or Aussie.mp. or exp Dominica/ or Dominica.mp. or Dominican.mp. or exp Slovenia/ or Slovenia.mp. or Slovene.mp. or Slovenian.mp. or (Trinidad and Tobago).mp. or exp "Trinidad and Tobago"/ or "Trinidad and Tobago".mp. or Trinidadian.mp. or Tobagonian.mp. or Trinis.mp. or Trinbagonian.mp. or exp Lithuania/ or Lithuania.mp. or Lithuanian.mp. or exp Barbados/ or Barbados.mp. or Barbadian.mp. or Bajan.mp. or (Saint Helena or Saint Helenian).mp. or exp Brazil/ or Brazil.mp. or Brazilian.mp. or exp Netherlands/ or Netherlands.mp. or Dutch.mp. or Nederlander.mp. or Holland.mp. or exp Azerbaijan/ or Azerbaijan.mp. or Azerbaijani.mp. or exp Curacao/ or Curacao.mp. or Curacaoan.mp. or (Sint Eustatius and Saba).mp. or (Bonaire or St Eustatius or Statia or Statian or BES Island*).mp. or (Sint Eustatius and Saba).mp. or exp United States/ or United States.mp. or America.mp. or American.mp. or USA.mp. or "U.S.A".mp. or "the US".mp. or "U.S.".mp. or (Turks and Caicos Islands).mp. or ("Turks and Caicos Island*" or "Turks and Caicos Islander*").mp. or exp Estonia/ or Estonia.mp. or Estonia.mp. or exp Sweden/ or Sweden.mp. or Swede.mp. or Swedish.mp. or exp Montenegro/ or Montenegro.mp. or Montenegrin.mp. or exp Iran/ or Iran.mp. or Iranian.mp. or Persian.mp. or exp Polynesia/ or Polynesia.mp. or Polynesian.mp. or exp Czech Republic/ or Czechia.mp. or Czech.mp. or Czechic.mp. or exp Indian Ocean Islands/ or Maldives.mp. or Maldivian.mp. or exp Colombia/ or Colombia.mp. or Colombian.mp. or exp Denmark/ or Denmark.mp. or Danes.mp. or Danish.mp. or exp Iceland/ or Iceland.mp. or Icelander.mp. or Icelandic.mp. or exp Romania/ or Romania.mp. or Romanian.mp. or Rumanian.mp. or Roumanian.mp. or exp Ireland/ or Ireland.mp. or Irish.mp. or Irelander.mp. or Irishman.mp. or exp Brunei/ or Brunei.mp. or Bruneian.mp. or exp New Zealand/ or New Zealand.mp. or New Zealander.mp. or exp France/ or France.mp. or French.mp. or Frenchman.mp. or Frenchwoman.mp. or (Vincent and the Grenadines).mp. or exp "Saint Vincent and the Grenadines"/ or "Saint Vincent and the Grenadines".mp. or Vincentian.mp. or "Vincent and the Grenadines".mp. or exp Moldova/ or Moldova.mp. or Moldovan.mp. or exp Qatar/ or Qatar.mp. or Qatari.mp. or exp El Salvador/ or El Salvador.mp. or Salvadoran.mp. or Salvadorian.mp. or exp Bahrain/ or Bahrain.mp. or Bahrani.mp. or exp "Democratic People's Republic of Korea"/ or North Korea.mp. or People's Republic of Korea.mp. or North Korean.mp. or Korean.mp. or DPRK.mp. or exp Mexico/ or Mexico.mp. or Mexican.mp. or exp Gibraltar/ or Gibraltar.mp. or Gibraltarian.mp. or exp Argentina/ or Argentina.mp. or Argentinian.mp. or Argentine.mp. or exp Turkey/ or Turkey.mp. or Turkish.mp. or exp Martinique/ or Martinique.mp. or Martiniquais.mp. or Martinican.mp. or exp Cabo Verde/ or Cape Verdean.mp. or Cabo Verdean.mp. or (Wallis and Futuna Islands).mp. or exp "Wallis and Futuna Islands"/ or Wallisian.mp. or Futunan.mp. or "Wallis and Futuna".mp. or (Wallis and Futuna Islands).mp. or exp Vietnam/ or VietNam.mp. or Vietnam.mp. or Vietnamese.mp. or Viet.mp. or exp Greenland/ or Greenland.mp. or Greenlandic.mp. or exp Sri Lanka/ or Sri Lanka.mp. or Sinhalese.mp. or Sri Lankan.mp. or exp Bangladesh/ or Bangladesh.mp. or Bangladeshi.mp. or Bengali.mp. or exp Grenada/ or Grenada.mp. or Grenadian.mp. or exp Belize/ or Belize.mp. or Belizean.mp. or exp New Caledonia/ or New Caledonia.mp. or Kanak.mp. or Kanaky.mp. or exp Ecuador/ or Ecuador.mp. or Ecuadorian.mp. or exp India/ or India.mp. or Indian.mp. or Hindu.mp. or exp Nepal/ or Nepal.mp. or Nepali.mp. or Nepalese.mp. or exp Kuwait/ or Kuwait.mp. or Kuwaiti.mp. or exp Guadeloupe/ or Guadeloupe.mp. or Guadeloupean.mp. or exp "Georgia (Republic)"/ or Georgia.mp. or Georgian.mp. or exp Lebanon/ or Lebanon.mp. or Lebanese.mp. or exp Tunisia/ or Tunisia.mp. or Tunisian.mp. or exp Asians/ or exp Europe/ or exp Europe, Eastern/ or exp European Union/ or exp Asia/ or exp Asia, Southeastern/ or exp Asia, Central/ or exp Asia, Western/ or exp Asia, Northern/ or exp Americas/ or exp Caribbean Region/ or exp Central America/ or exp Latin America/ or exp North America/ or exp South America/)  **Restricted to articles published between 2000 and 2022 and removed conference abstracts** | 5 |

## Table S2. Inclusion and exclusion criteria.

|  | **Inclusion criteria** | **Exclusion criteria** |
| --- | --- | --- |
| **Study design** | - Cross-sectional studies - Longitudinal studies - Intervention studies - Qualitative studies | - Abstracts - Conference proceedings - Letters - Commentaries - Editorials - Reviews - Preprints |
| **Population** | - Nulliparous women - Women with at least post-secondary education (above 12 years of schooling)^*^ - Women of reproductive age (15-49 years) | - Women who already have children or are pregnant - Women with below post-secondary education^*^ - Women below 15 years or above 49 years old |
| **Outcome** | - Fertility intentions (intention not to have children) - Perceived conditions for motherhood | None |
| **Language** | All language | None |
| **Time period** | 2000 to 2022 | Articles published before 2000 or after the initial of our study |
| **Country** | 125 countries with a total fertility rate below 2.1 births per woman | Countries with a total fertility rate higher than 2.1 births per woman |

^*^Post-secondary education includes universities, (community) colleges, vocational schools, and technical training institutes.

## Table S3. Risk of bias assessment for eligible studies.

| **Category** | **Studies reported fertility intentions** | **Studies reported factors associated with fertility intentions** |
| --- | --- | --- |
| **Total** | 27 (100.0%) | 15 (100.0%) |
| **Overall quality** |  |  |
| Low risk (low risk for all criteria) | 6 (22.2%) | 1 (6.7%) |
| High risk (high risk in at least one criterion) | 15 (55.6%) | 7 (46.7%) |
| Moderate risk (some concerns in at least one criterion) | 6 (22.2%) | 7 (46.7%) |
| **Representativeness** |  |  |
| Low risk | 23 (85.2%) | 11 (73.3%) |
| High risk | 4 (14.8%) | 4 (26.7%) |
| Unclear | 0 (0.0%) | 0 (0.0%) |
| **Sample size** |  |  |
| Low risk | 21 (77.8%) | 6 (40.0%) |
| High risk | 6 (22.2%) | 1 (6.7%) |
| Unclear | 0 (0.0%) | 8 (53.3%) |
| **Non-response rate** |  |  |
| Low risk | 10 (37.0%) | 5 (33.3%) |
| High risk | 9 (33.3%) | 4 (26.7%) |
| Unclear | 8 (29.6%) | 6 (40.0%) |
| **Data collection tool** |  |  |
| Low risk | 27 (100.0%) | 14 (93.3%) |
| High risk | 0 (0.0%) | 1 (6.7%) |
| Unclear | 0 (0.0%) | 0 (0.0%) |

## Table S4. Sources of heterogeneity assessed using meta-regression.

| **Moderator** | **Univariate meta-regression Residual** $\boldsymbol{I}^{\boldsymbol{2}}$ | **Univariate meta-regression** $\boldsymbol{R}^{\boldsymbol{2}}$ | **Univariate meta-regression *p*-value** | **Multivariate meta-regression residual** $\boldsymbol{I}^{\boldsymbol{2}}$ | **Multivariate meta-regression** $\boldsymbol{R}^{\boldsymbol{2}}$ | **Multivariate meta-regression *p*-value** |
| --- | --- | --- | --- | --- | --- | --- |
| Study location | 97.65% | 21.66% | < 0.0001 | 97.08% | 6.48% | < 0.0001 |
| Income classification | 98.22% | 3.03% | < 0.0001 |  |  |  |
| Study period | 98.30% | 0.00% | < 0.0001 |  |  |  |
| Age of participants | 98.48% | 0.81% | < 0.0001 |  |  |  |
| Recruitment setting | 98.55% | 0.00% | < 0.0001 |  |  |  |
| Study type | 98.52% | 0.00% | < 0.0001 |  |  |  |
| Sample size | 98.59% | 0.00% | < 0.0001 |  |  |  |
| Leave policy | 97.74% | 5.42% | < 0.0001 |  |  |  |

^*^ Data on income classification and leave policies for each country during the respective study periods were collected from the World Bank (1, 2).

## Table S5. Egger’s regression test for funnel plot asymmetry assessment.

| **Test** | **Intercept** | **95% Confidence Interval** | **t** | ***p*-value** |
| --- | --- | --- | --- | --- |
| **Egger’s regression test** | 0.3389 | [0.22, 0.46] | 0.18 | 0.86 |

## Table S6. Factors associated with fertility intentions in quantitative studies.

| **Study title** | **Author** | **Study design** | **Sample size** | **Original description** | **Summary of relevant factors** | | | | |
| --- | --- | --- | --- | --- | --- | --- | --- | --- | --- |
|  |  |  |  |  | **Individual factors** | **Family factors** | **Community factors** | **Institutional factors** | **Structural factors** |
| Intentions and attitudes towards parenthood and fertility awareness among Chinese university students in Hong Kong: a comparison with Western samples | Chan (2015)(3) | Quantitative study | 275 | When asked about potential obstacles to parenthood, Hong Kong respondents were concerned with ‘not finding the correct partner’(68%), ‘pursuit of career aspirations’ (49%), ‘financial concerns’ (39%), ‘not feeling emotionally ready’ (33%), ‘educational pursuit’ (17%), ‘pursuit of personal interest’ (16%) and ‘infertility’ (3%). | pursuit of career aspirations | correct partners/stable relationship | / | / | / |
|  |  |  |  |  | financial security | / | / | / | / |
|  |  |  |  |  | mature/emotionally ready | / | / | / | / |
|  |  |  |  |  | pursuit of personal interest | / | / | / | / |
|  |  |  |  |  | education | / | / | / | / |
| Fertility and fertility preservation: knowledge, awareness and attitudes of female graduate students | Hickman (2018)(4) | Quantitative study | 1003 | The reasons cited for delaying childbearing were multifactorial, with career building noted most commonly (69%), followed by desire for financial security (50%) and need for a partner (35%). | pursuit of career aspirations | correct partners/stable relationship | / | / | / |
|  |  |  |  |  | financial security | / | / | / | / |
| Birth rate and fertility: knowledge and expectations analysis of 3585 university students | Machado (2014)(5) | Quantitative study | 2403 | Obtiveram-se respostas de 2403 estudantes quanto aos factores condicionantes da probabilidade de ter filhos cuja análise global permite concluir que ter emprego, o parceiro ter vontade de ter filhos, ter segurança financeira para proporcionar bons cuidados de saúde e uma boa edu_x005f cação aos filhos, ter estabilidade no casamento ou união de facto, assim como no emprego, são considerados muito importantes (mais de 30% atribuiu grau 1/2). Os factores considerados pouco importantes (grau 9/10) por mais de 30% dos respondentes são ter carro e ser casado ou unido de facto. Ter casa adequada e apoio de familiares foram considerados de importância média. | pursuit of career aspirations | correct partners/stable relationship | / | housing security | / |
|  |  |  |  |  | financial security | partner's fertility intention | / | labor market | / |
|  |  |  |  |  | / | family support | / | / | / |
|  |  |  |  |  | / | / | / | / | / |
| Childbearing Decisions in Residency: A Multicenter Survey of Female Residents | Stack (2020)(6) | Quantitative study | 274 | Self-reported determinants of delaying childbearing, which were not mutually exclusive. The most frequently reported factor was “busy work schedule” (n = 255; 93%), with one respondent commenting, “I don’t care at all about extending residency, but it would break my heart to go back to work after 6 weeks.” The next most frequently reported factors, in order, were “desire to not extend my residency training” (n = 145; 53%), “lack of access to childcare” (n = 126; 46%), and “finances” (n = 116; 42%). | pursuit of career aspirations | / | high workload | childcare services | / |
|  |  |  |  |  | financial security | / | / | / | / |
|  |  |  |  |  | education | / | / | / | / |
|  |  |  |  | Many respondents cited among their top 3 reasons for delaying childbearing the concern that “parental leave would burden my colleagues” (n = 96; 35%). Twenty-seven percent (n = 74) of respondents cited the concern that “residency training might increase pregnancy complications” as a reason for delaying childbearing. | / | / | parental leave burden on colleagues | / | / |
|  |  |  |  |  | / | / | / | / | / |
|  |  |  |  | There were 35 free-text responses submitted by the 274 respondents who indicated they were delaying childbearing. The categories resulting from the analysis of these responses included not feeling ready for parenthood (n = 19; 7%), lack of family support (n = 5; 2%), concerns of maintaining healthy pregnancy in residency (n = 5; 2%), lack of time at home (n = 3; 1%), and an unsupportive program (n = 1; 0.4%). Career threat (n = 2; 1%) was also mentioned both with respect to pregnancy, which was seen as a disadvantage for physicians interviewing for future opportunities, and to parenthood, which could negatively affect a physician in the early years of establishing a career. | not ready for parenthood | family support | / | / | / |
|  |  |  |  |  | lack of time at home | / | career threat | / | / |
|  |  |  |  |  | / | / | unsupportive program | / | / |
| Cross-sectional pregnancy survey on fertility trends and pregnancy knowledge in Singapore | Tan (2011)(7) | Quantitative study | 36 | Not married yet: 50% (18/36) Not prepared to change lifestyle: 33.3% (12/36) Financial difficulty: 8.3% (3/36) No family support to look after children: 2.8% (1/36) Other: 2.8% (1/36) Missing: 2.8% (1/36) | not prepared to change lifestyle | correct partners/stable relationship | / | / | / |
|  |  |  |  |  | financial security | family support | / | / | / |
| Female university students' attitudes to future motherhood and their understanding about fertility | Tydén (2006)(8) | Quantitative study | - | The reasons given in the free text were: could not take responsibility for any other persons; were afraid of being a bad parent; or disliked children. | mature/emotionally ready | / | / | / | / |
|  |  |  |  |  | not ready for parenthood | / | / | / | / |
|  |  |  |  |  | fear of childrearing |  |  |  |  |
|  |  |  |  |  | dislike children | / | / | / | / |
| Fertility Intentions, Parenting Attitudes, and Fear of Childbirth among College Students in China: A Cross-Sectional Study | Xu (2022)(9) | Quantitative study | - | Responders who did not want children and those who were unsure about having children had higher levels of fear (mean = 48.80, SD = 8.03) than those who wanted children (mean = 47.16, SD = 7.70; t = −2.439, P < .02). | Childbirth Fear Prior to Pregnancy (CFPP) | / | / | / | / |
| Fertility intentions among young people in the era of China's three-child policy: a national survey of university students | Zhang (2022)(10) | Quantitative study | - | Age: β=0.100 (p<0.001) Current study grade: β=-0.031 (p=0.201) Monthly household income: β=-0.002 (p=0.886) Paternal highest educational level: β=0.001 (p=0.964) Maternal highest educational level: β=-0.017 (p=0.391) Knowledge about reproductive, maternal, newborn, and child health (RMNCH) support and/or services: β=0.068 (p<0.001) Parenthood–related anxiety: β=-0.151 (p<0.001) Childbearing- and childbirth-related anxiety: β=-0.119 (p<0.001) | Age | / | / | / | / |
|  |  |  |  |  | Knowledge about reproductive, maternal, newborn, and child health (RMNCH) support and/or services | / | / | / | / |
|  |  |  |  |  | psychological stress | / | / | / | / |
| Family planning among female medical students: are their plans comparable to other professionals? | Araujo (2020)(11) | Quantitative study | 155 | Career: 40% (4/10) Medical student; 20% (2/10) Law student; Not a personal project/wish: 40% (4/10) Medical student; 70% (7/10) Law student; Did not answer: 20% (2/10) Medical student; 10% (1/10) Law student; | pursuit of career aspirations | correct partners/stable relationship | / | / | / |
|  |  |  |  |  | pursuit of personal interest | / | / | / | / |
|  |  |  |  | For both groups, the priorities were financial planning and professional career, but specialization/post-graduation were cited only among MS (16 citations). Family structure was mentioned as an important issue, as well as maternal age/maternal health (cited by approximately a third and half of the cohorts, respectively).  There were notable differences in a comparison between MS that would choose a surgical specialty (NSMS–73 students, 77.8%) and other MS (SMS–21 students–22.3%). Concerning the reasons influencing familial planning, there were notable differences among NSMS and SMS. Career and specialization were much more cited as important by SMS (p=0.053 and p=0.001, respectively). Surprisingly, both cohorts denied family planning would affect professional choices in similar proportions (p=0.831). Family structure, in contrast, was much more important for NSMS. | maternal age | / | / | / | / |
|  |  |  |  |  | financial security | / | / | / | / |
|  |  |  |  |  | Education (major) | / | / | / | / |

## Table S7. Factors associated with fertility intentions in qualitative and mixed-method studies.

| **Study title** | **Author** | **Study design** | **Sample size** | **Original description** | **Summary of relevant  factors** | | | | |
| --- | --- | --- | --- | --- | --- | --- | --- | --- | --- |
|  |  |  |  |  | **Individual factors** | **Family factors** | **Community factors** | **Institutional factors** | **Structural factors** |
| Factors Influencing Women’s Decisions About Timing of Motherhood | Benzies (2006)(12) | Qualitative study | 45 | Women who decided to have their children later in life were more likely than women who had their children earlier to stress the importance of establishing independence through education, secure employment, and financial stability. Catherine who was in her late 30s without children stated:  "My mom always stressed that I needed to support myself and be independent. I went and got a practical degree and got a career started. It wasn’t until I finished my degree that I was even able to consider getting married, so there was a lot of pressure [about getting an education] there." | education | / | / | / | / |
| Attitudes towards family formation in cohabiting and single childless women in their mid- to late thirties | Birch Petersen (2016)(13) | Qualitative study | 20 | There was a feeling of ‘a point of no return’ and one woman felt as if she was ‘held at gunpoint’ (Sanne, 37). The women were aware of the age-related decline in female fecundity and clearly expressed that the decision view that having children was becoming increasingly urgent. The ‘biological clock’ in combination with a wish of fulfilling social norms, were perceived as strenuous and resulted in feeling guilty of not being broody. It became a conflict of choosing. Currently, there is some debate that we are in fact too old when we have children, and that this is especially true for the academic women. | age | / | / | / | societal norm |
|  |  |  |  | If women choose to have children, they have to deselect other things. These concerns were surprisingly similar irrespective of relationship status and educational level. All of the women, except one, had a vocational training of three to six years and all were employed. Their lives had been occupied by education, careers and travelling. ‘‘The correct order is: education, job, and children and in addition to have a stable financial situation. To get the feeling that you have established that ‘‘package’’, which you safely and soundly can fit a child into. When you are living haphazardly – or not haphazardly – but perhaps a bit more irresponsible, then it is like – since things did not happen, I have to change the order a bit – or at least, that is what I’m doing.’’ (Caroline, 34) | education | correct partners/stable relationship | / | / | / |
|  |  |  |  |  | career | / | / | / | / |
|  |  |  |  |  | pursuit of personal interest | / | / | / | / |
|  |  |  |  |  | financial security | / | / | / | / |
|  |  |  |  | The timing of a pregnancy was important and had to be ‘right’. Despite the women’s advanced age they did not feel ‘ready’ to become a mother and only a few addressed being ‘broody’ – in fact most mentioned the opposite.  ‘‘I have never had that feeling of being broody, so I guess, that it is something I want to do, so I don’t end up regretting not getting children later on.’’(Cathrine, 37) | mature/emotionally ready | / | / | / | / |
|  |  |  |  | Sometimes, when I read some of those articles, I feel hit at a sore spot and I think: ‘‘Well – I would have liked to have children, had I only met that someone’’; it is presented as though you are extremely picky, but it is not like that. I’m not sure if I feel pressured, perhaps sometimes you do feel hurt.’’(Sarah, 38) ‘‘For many years, I have been searching for a man with whom I could have children with. I haven’t succeeded in finding the one with whom I could start a family, so now I’m considering whether to do things in a different order.’’ (Sally, 38) | / | correct partners/stable relationship | / | / | / |
|  |  |  |  | ‘‘What you really want, is a family and children. But, it is not as easy as some make it sound.’’ (Signe, 35) Finding the right man is difficult and the next step– ‘is he ready to have children’ was also an issue. The women were frustrated because they had a feeling that men were holding them back. Despite the women’s concerns of having children they were more resolved and expressed themselves more ‘ready’ than their male partners. | / | partner's fertility intention | / | / | / |
|  |  |  |  | The women had a picture of ‘The good mother’ as a woman in control over her life with an emphasis on the family. The women experienced personal and society-induced expectations in terms of: the perfect mother, wife, friend, colleague, cook, etc., and intended to pursue this. | / | / | / | / | societal norm |
| The ABC of reproductive intentions: a mixed-methods study exploring the spectrum of attitudes towards family building | Grace (2022)(14) | Mixed-method study | 20 | ‘Okay, I have never, ever, ever wanted children. You know? I just never ever wanted children of my own.... No, I just never ever thought, oh, I would love to have a child someday. I just never had that maternal instinct’. FP8—Female, Age 28, White, Degree qualification, has no child, no desire for children. | dislike children | / | / | / | / |
|  |  |  |  | ‘Having children is environmentally irresponsible, you know; the world is really overpopulated already and stuff and I don’t think most people are aware of that or that it governs people’s choices’. FP9—Female, Age 33, White, Degree qualification, no child, no desire for children. | / | / | / | / | environment (global warming/climate) |
| Exploring the perception of childbearing barriers in a low fertility subgroup of Iran: a qualitative study | Safari-Faramani (2018)(15) | Qualitative study | 11 | Most of the participants complained about high mental duress, high workload, and insufficient time for childcare. Both men and women believed that educated groups are so busy that they cannot allocate time for thinking about childbearing. Furthermore, they talked about intrinsic interest in childbearing, and emphasized that a competitive atmosphere should have profited women. Also, they demanded a decrease in the duration of working hours of women or increased annual leave for mothers. A 40-year-old woman, a faculty member with no children said:“…Once I was talking with one of my colleagues, who is a psychologist herself. I said I didn’t feel the absence of a child in my life so I didn’t think of having one. That is because I’m really busy, maybe there should be more free time for faculty members, as we are too busy…” | psychological stress | / | high workload | labor market (working hour, annual leave) | / |
|  |  |  |  |  | / | / | work competition | / | / |
|  |  |  |  |  | / | / | incompatibility between work and family | / | / |
|  |  |  |  | Preferring social roles over maternal roles among girls: Some of the participants pointed out that since our mothers had many children, they always held this idea that a social position is much desirable for girls than childbearing. A 32-year-old woman, a PhD student with no children said: “…In the past, there was this saying that a girl will be a babysitter finally, but our moms answered strictly that their daughter would not be a babysitter, she would be a doctor or engineer someday and take a nanny to care for her children…”(p-11). Some of the participants complained that our social structures are not proper for training mothers and fathers. Also, the heavy competitive atmosphere prevailing in the educational system is not designed to educate mothers and fathers. | / | / | / | / | societal norm |
|  |  |  |  | On the other hand, as the participants maintained, educated couples who marry late may want to spend time for getting to know each other and creating a stable financial situation. Since they don’t have enough time for childbearing, they stop at one. A 32-year-old woman, a PhD student, with no children, said: “…My husband says: we don’t want children until our life becomes stable, and of course we need to know if we can get along with each other before bringing another person into this world, we need to match with each other since we are newly married…” (p - 11). | / | correct partners/stable relationship | / | / | / |
| Postindustrial fertility ideals, intentions, and gender Inequality: a comparative qualitative analysis | Brinton (2018)(16) | Qualitative study | 207 | In Spain and Sweden, financial concerns were generally expressed in terms of the importance of securing a stable job. This mirrors research on the two countries that mentions the norm of achieving a stable income before having children (Ahn and Mira 2001; Andersson 2002). In Sweden, wage replacement during parental leave is currently set at 80 percent, but only permanent workers are guaranteed the right to return to the same or a similar job (Oláh and Bernhardt 2008). This factor constitutes a strong incentive to secure a stable, well-paying job before having a child. The interpretations offered by our Spanish interviewees for their fertility intentions and the ideals/intentions gap, together with their actual employment situation as shown in Table 2, illustrate the high level of labor market uncertainty in Spain. Characteristics of our Spanish sample are consistent with quantitative data showing that economic insecurity is a major phenomenon even among highly educated young adults (OECD 2017). | financial security | / | / | labor market (parental leave and wage replacement) | economy |
|  |  |  |  | For instance, educational costs were raised exclusively by Japanese and American interviewees. This is not surprising, given that in Spain and Sweden free primary and secondary education is guaranteed and college education is very affordable. The absence of this concern among Swedish interviewees likely reflects the fact that Sweden offers high-quality state-subsidized childcare and paid childcare leave (Oláh and Bernhardt 2008), whereas the US and Spain lack such policies. Many Japanese interviewees expressed worry over the long waiting lists in urban areas for public childcare, anticipating that this might make it necessary to pay a substantial amount for private childcare or to rely on grandparents. Interviewees stressed that high-quality childcare is difficult to find and, when available, very costly. This leads some of the American women in full-time dual-earner couples to anticipate leaving the labor force to become stay-at-home mothers for at least some period of time. Similar to Spanish interviewees’ reasoning, Swedish interviewees often raised concerns about not having a stable full-time job, although in Sweden this was mentioned by many more women than men (in contrast with concern among both sexes in Spain). As mentioned earlier, this may reflect Sweden’s generous parental leave scheme, which provides an incentive for women to secure a well-paying job before becoming pregnant. | / | / | / | education services | / |
|  |  |  |  |  | / | / | / | childcare services | / |
|  |  |  |  |  | career/employment | / | / | maternity benefits | / |
|  |  |  |  | Similarly, Japanese women who expressed their commitment to full-time work often had higher fertility ideals than intentions, saying that they simply would not be able to “have it all.” This reflected their perception of the likely impossibility of combining full-time employment and childrearing, especially for more than one child. | / | / | incompatibility between work and family | / | / |
|  |  |  |  | In sum, instead of explicitly questioning the highly gender-unequal household division of labor or citing it as a reason for lower fertility intentions, nearly all Japanese female interviewees took gender specialization in the household as a given. This tended to translate into lower fertility intentions in couples where the wife worked full-time. The effect of labor market conditions on the fertility goals of highly educated Japanese interviewees asserts itself instead through long working hours, which limit men’s availability for housework and childcare and pose an either/or decision between employment and childbearing for many married women. Coupled with the rapid changes in gender-role ideology in Spain discussed earlier, the Spanish economic context pushes couples strongly toward a dual-earner–dual-carer model. We suspect that the embrace of this model is one reason for the higher fertility intentions of our Spanish than Japanese interviewees. Another line of reasoning that we heard from some Spanish males but no Japanese males was that the couple might consider having more children if he commits more time to household labor and childcare. Some Spanish female interviewees indeed express their desire for this outcome, in contrast to Japanese female interviewees’ implicit acceptance of a highly skewed household division of labor. The majority of Swedish male interviewees had permanent job contracts, and the rest were either self-employed or in a temporary contract but working full-time. In contrast, fewer than half of Swedish female interviewees were in jobs with a permanent contract; almost as many had temporary contracts, and the remainder were self-employed or unemployed. A number of Swedish female interviewees also expressed worries about the negative attitude some employers have toward women with care responsibilities, and voiced fears of becoming caught in the “part-time [employment] trap.” Some also voiced concern over whether it would take so long to acquire a stable job that they would “run out of time” to become pregnant. most American male and female interviewees state their assumption that women will be the primary caregivers. Many American female interviewees say they do not expect their husbands to share childrearing equally or to adapt their employment to parenthood. Among women who plan to continue working full-time, many expressed worry about potential work/family conflict and the risk of incurring a motherhood wage penalty. | career/employment | housework responsibility | work competition | labor market (working hour) | societal norm (gender inequality) |
|  |  |  |  |  | / | / | social pressure (prejudice towards pregnancy from employers) | / | / |
|  |  |  |  | The work/family conflict expressed by American female interviewees is consistent with the emphasis of gender equity theory not only on the importance of gender egalitarianism in the household division of labor but also on the support of social institutions and policies for combining work and family. The lack of such institutional support for American dual-earner families is reflected in interviewees’ (especially women’s) perceptions of how difficult and expensive the work/family balancing act is likely to be. | / | / | / | / | national policies |
| Opportunity costs and Latinas' family formation attitudes | Spence (2016)(17) | Qualitative study | 11 | . . . I’ve heard this from a lot of South American and Central American friends. Their fathers are in control of their lives . . . “Stay at home.” “Watch your brothers and sisters.” “Get married.” “Have this child.” They plan it out for them. (Jessica) Less specific references to culture also tended to allude to gendered expectations, particularly women’s responsibility for the domestic sphere. | / | / | / | / | societal norm |
|  |  |  |  | I think as time progresses . . . the Hispanic community—especially coming to America—I think they will have less children because the economy in the Dominican Republic isn’t that great. So people are automatically having less children, but my aunt they had another child, a third child. It’s a blessing, but “ehh, do we have the funds to have another child?” So, I do think that they’ll have less children in the Hispanic community. (Delilah) | / | / | / | / | immigration |
|  |  |  |  | I think religion plays a big part with the family size, but the way the economy and the society is doing can easily trump religion because women are supposed to—well they are taught—to stay at home and cook. But there are a lot of children that are raised and their fathers aren’t there, so then whatever the church is saying goes out the window because the mother has to provide for her children. (Delilah) | religion | / | / | / | economy |
|  |  |  |  | I agree with Lily, ’cause you have to be mentally, financially prepared to have children. You want to ’cause once you have children and you haven’t finished school, it just derails you, your timeframe to go back. There are lots of people who have gone back to school while they were raising their children, and I give them credit for that. But it’s not the same, when you’re mentally focused, you’re determined to finish school then you have kids.. . . (Claudine) | education | / | / | / | / |
|  |  |  |  |  | mature/emotionally ready | / | / | / | / |
|  |  |  |  |  | financial security | / | / | / | / |
|  |  |  |  | I think a lot of my friends haven’t influenced me on how many children I want to have, but they have influenced me that I do want to get educated and get a good job because most of my friends from high school didn’t go to college. They just, right away, after high school had children. . . . I can see them, and I feel that I don’t want to put them in a bad position, but I feel fortunate to not have children. It’s not just expensive; it takes away so much time from your life. You don’t enjoy life the same. Other study participants referenced generational changes in thinking about the opportunity costs of having children. However, their goals were less specifically career focused. Instead, Delilah and Lily describe how having children will limit their ability to travel the world. Delilah, a young childfree woman, describes this in a recent conversation with her mother who is an immigrant from the Dominican Republic: I always think that they came to this country to give me a better life. So the other day, I was joking with her, saying I was going to go travel the world, and she was like “how you’re going to have money for that?” And I said I wouldn’t have children. You had children, and you can’t travel. I think she was a bit offended . . . (Delilah) | pursuit of personal interest | / | / | / | generational changes |

# Figures

Figure S1. Within-region variations in the proportion of highly educated women who intend not to have children.

**
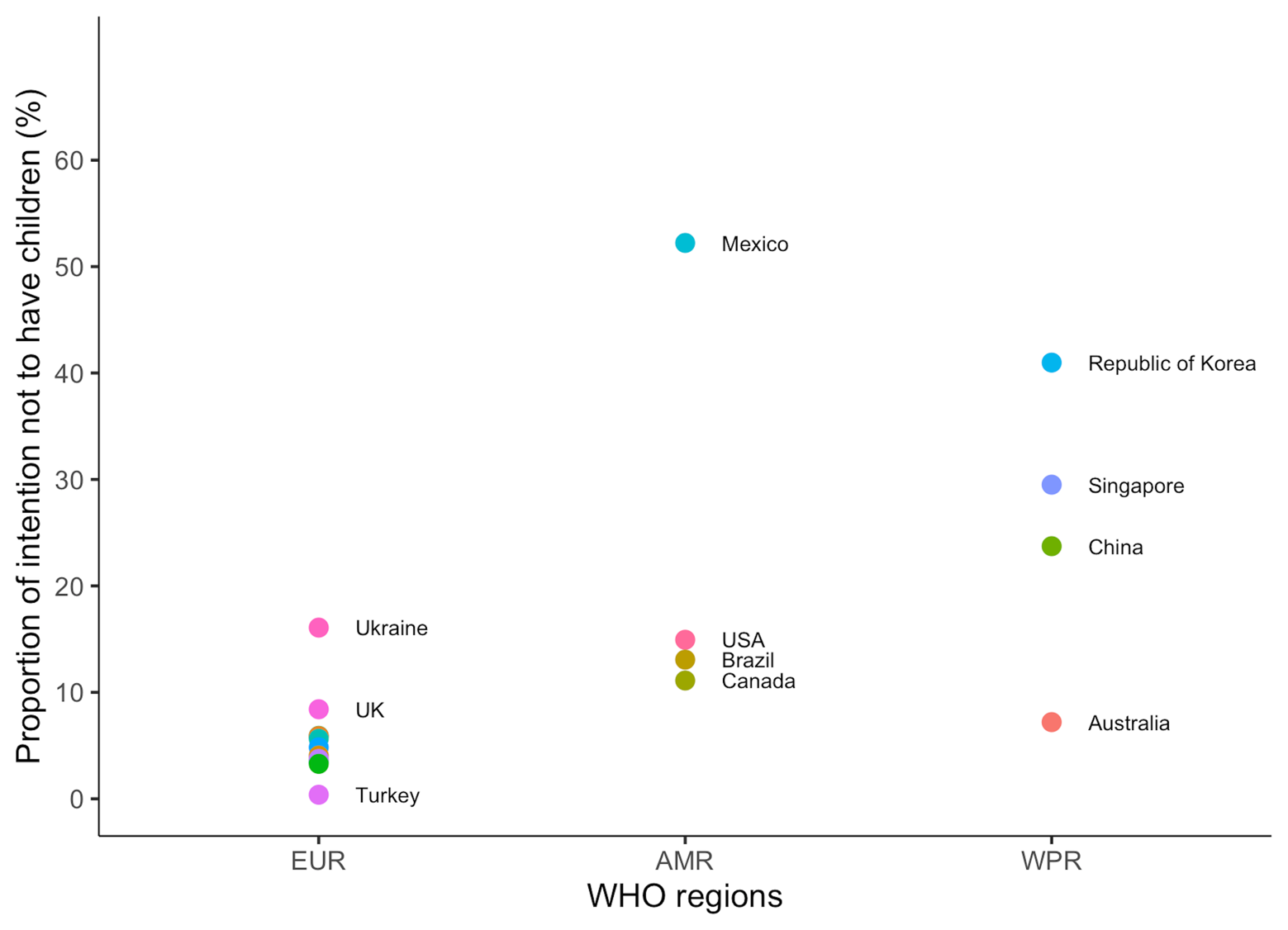
**

Abbreviations: UK = United Kingdom, USA = United States of America, EUR = European Region, AMR = Region of the Americas, WPR = Western Pacific Region, WHO = World Health Organization.

Figure S2. Sensitivity analysis using leave-one-out analysis. To evaluate the impact of individual studies on the synthesized result of the meta-analysis, we conducted a sensitivity analysis by excluding one study at a time. The results indicated that our meta-analysis was robust and not reliant on any single study. After a study was omitted, the proportion of intention not to have children varied between 11.12% (95% CI 7.78-14.97%) and 13.13% (9.28-17.53%).


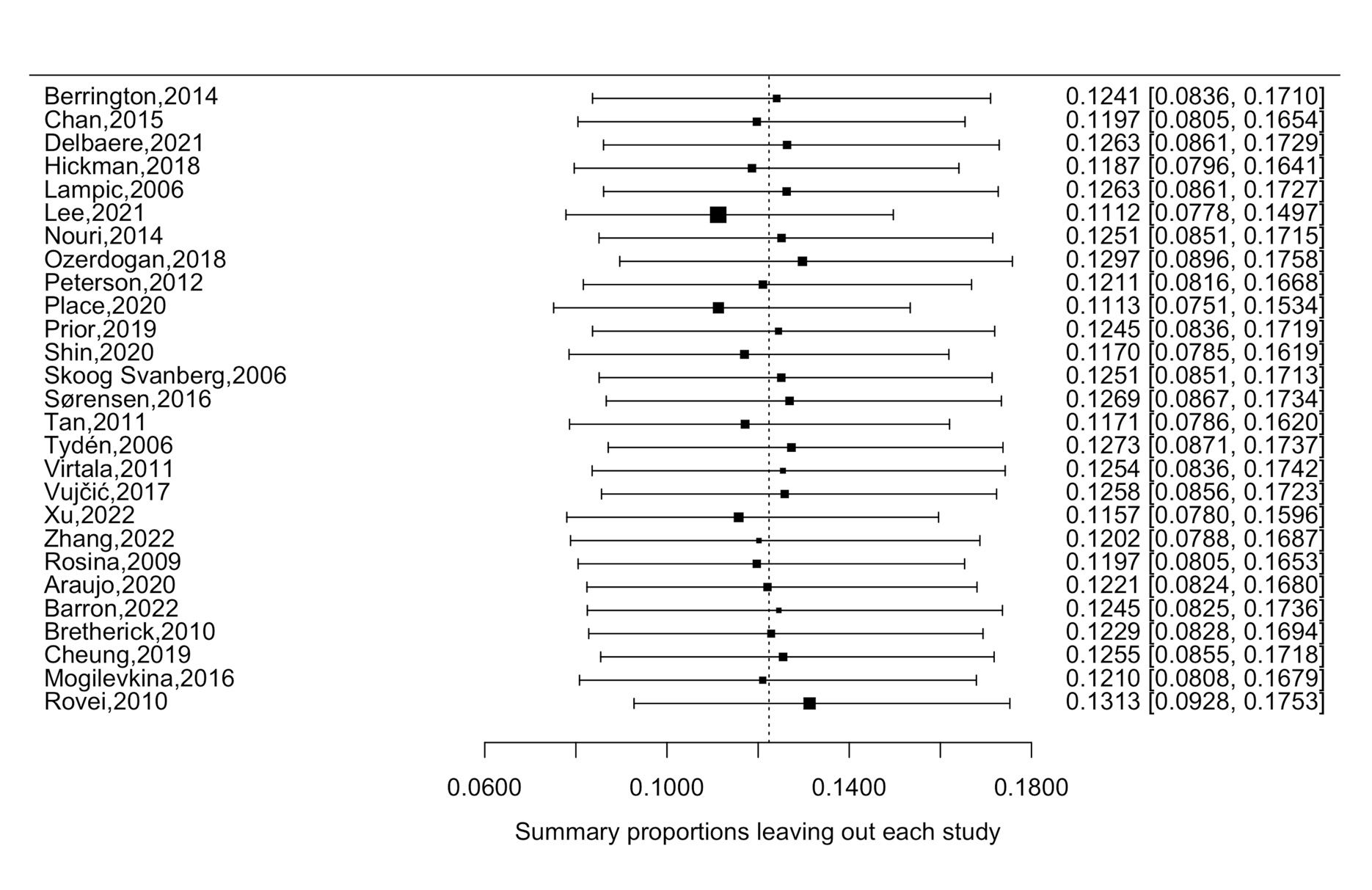


## Figure S3. Funnel plot for publication bias assessment.


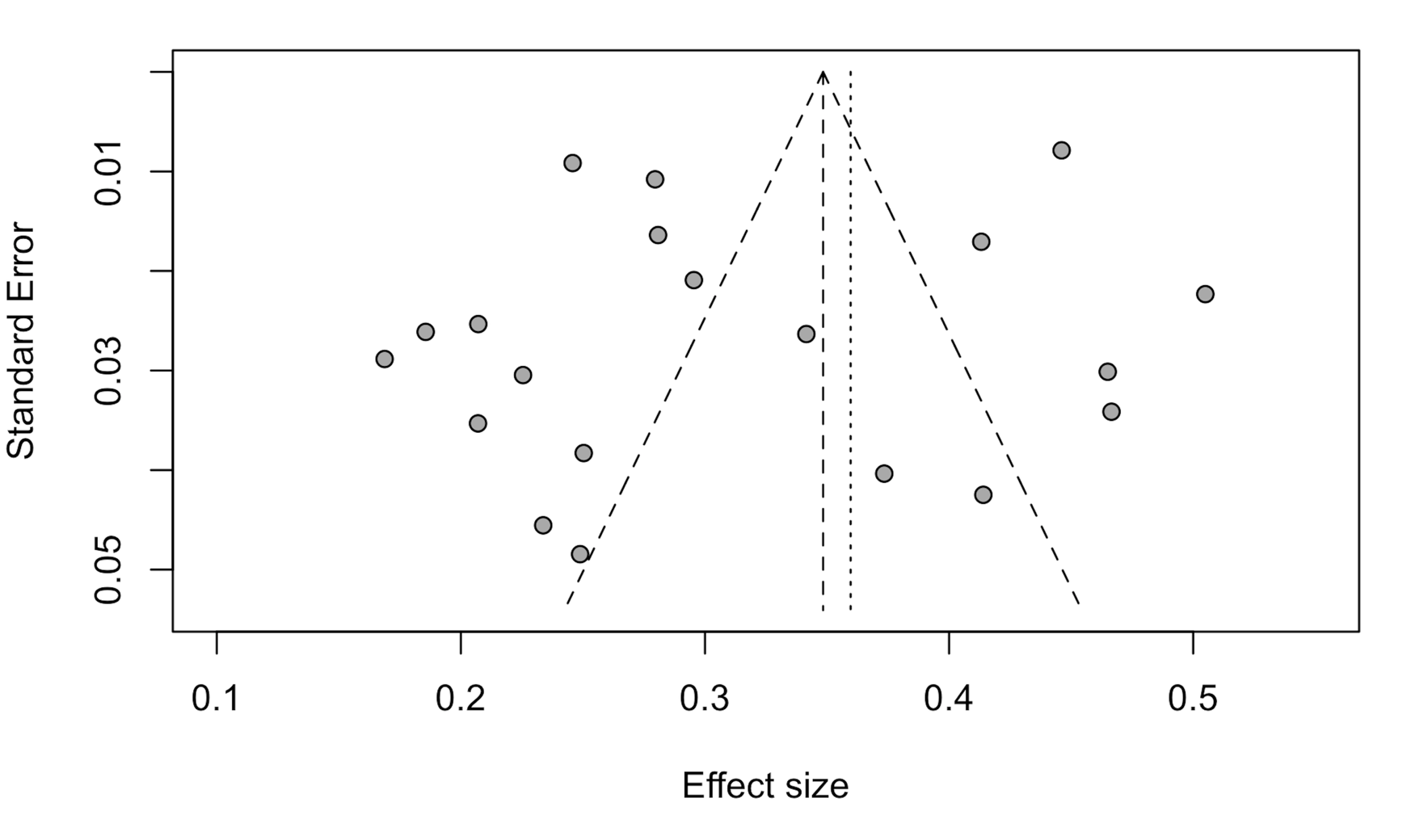


## Figure S4. Social-ecological model of factors associated with fertility intentions.

**
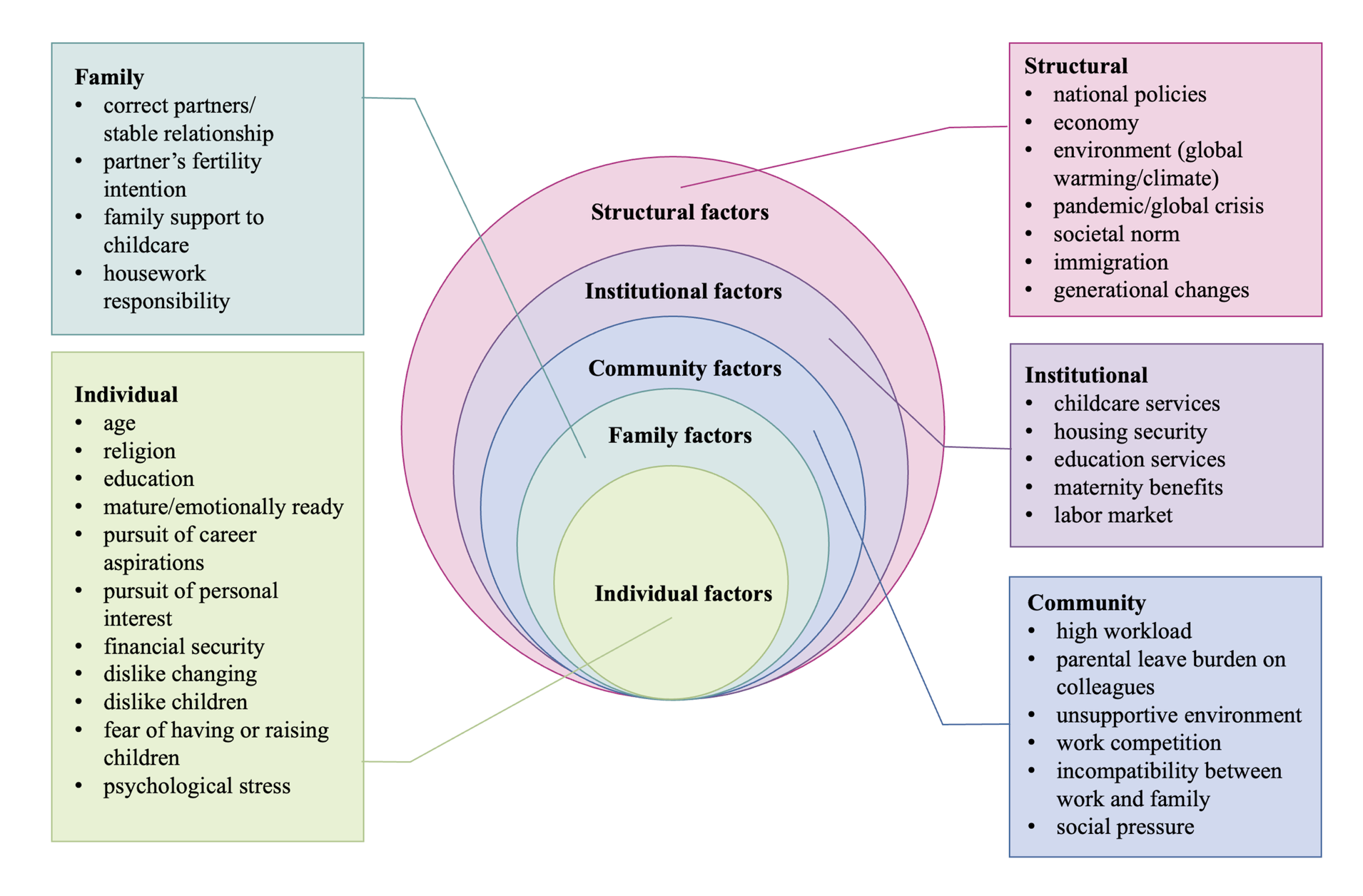
**

# Supplementary References

1. Women, Business and the Law. In: World Bank, editor.: World Bank,; 2024.

2. Income classification by country, 1987-2023. In: World Bank, editor.: World Bank,; 2024.

3. Chan CHY, Chan THY, Peterson BD, Lampic C, Tam MYJ. Intentions and attitudes towards parenthood and fertility awareness among Chinese university students in Hong Kong: a comparison with Western samples. Human reproduction (Oxford, England). 2015;30(2):364-72.

4. Hickman LC, Fortin C, Goodman L, Liu X, Flyckt R. Fertility and fertility preservation: knowledge, awareness and attitudes of female graduate students. The European journal of contraception & reproductive health care : the official journal of the European Society of Contraception. 2018;23(2):130-8.

5. Machado MdC, Alves MI, Couceiro L, Silva FGd, Morais de Almeida M, Alves I. [Birth rate and fertility: knowledge and expectations analysis of 3585 university students]. Natalidade e Fertilidade: Analise dos Conhecimentos e Expectativas de 3585 Estudantes Universitarios Portugueses. 2014;27(5):601-8.

6. Stack SW, Jagsi R, Biermann JS, Lundberg GP, Law KL, Milne CK, et al. Childbearing Decisions in Residency: A Multicenter Survey of Female Residents. Academic medicine : journal of the Association of American Medical Colleges. 2020;95(10):1550-7.

7. Tan TC, Tan SQ, Wei X. Cross-sectional pregnancy survey on fertility trends and pregnancy knowledge in Singapore. The journal of obstetrics and gynaecology research. 2011;37(8):992-6.

8. Tyden T, Svanberg AS, Karlstrom P-O, Lihoff L, Lampic C. Female university students' attitudes to future motherhood and their understanding about fertility. The European journal of contraception & reproductive health care : the official journal of the European Society of Contraception. 2006;11(3):181-9.

9. Xu J, Li L, Ma X-Q, Zhang M, Qiao J, Redding SR, et al. Fertility Intentions, Parenting Attitudes, and Fear of Childbirth among College Students in China: A Cross-Sectional Study. Journal of pediatric and adolescent gynecology. 2022.

10. Zhang C, Wei L, Zhu Y, Teng L, Zhang W, Xu J, et al. Fertility intentions among young people in the era of China's three-child policy: a national survey of university students. BMC pregnancy and childbirth. 2022;22(1):637.

11. Araujo J, Bacelar S, Jesus LE. Family planning among female medical students: are their plans comparable to other professionals? Rev Assoc Med Bras (1992). 2020;66(4):485-90.

12. Benzies K, Tough S, Tofflemire K, Frick C, Faber A, ra, et al. Factors influencing women's decisions about timing of motherhood. Journal of obstetric, gynecologic, and neonatal nursing : JOGNN. 2006;35(5):625-33.

13. Birch Petersen K, Sylvest R, Nyboe Andersen A, Pinborg A, Westring Hvidman H, Schmidt L. Attitudes towards family formation in cohabiting and single childless women in their mid- to late thirties. Human fertility (Cambridge, England). 2016;19(1):48-55.

14. Grace B, Shawe J, Johnson S, Usman NO, Stephenson J. The ABC of reproductive intentions: a mixed-methods study exploring the spectrum of attitudes towards family building. Human reproduction (Oxford, England). 2022;37(5):988-96.

15. Safari-Faramani R, Haghdoost AA, Baneshi MR, Dehnavieh R. Exploring the perception of childbearing barriers in a low fertility subgroup of Iran: a qualitative study. Electronic physician. 2018;10(6):6927-34.

16. Brinton MC, Bueno X, Olah L, Hellum M. Postindustrial fertility ideals, intentions, and gender Inequality: a comparative qualitative analysis. Population and Development Review. 2018;44(2):281-309.

17. Spence NJ. Opportunity costs and Latinas' family formation attitudes. Hispanic Journal of Behavioral Sciences. 2016;38(2):186-205.
